# Engineering transient dynamics of artificial cells by stochastic distribution of enzymes

**DOI:** 10.1038/s41467-021-27229-0

**Published:** 2021-11-25

**Authors:** Shidong Song, Alexander F. Mason, Richard A. J. Post, Marco De Corato, Rafael Mestre, N. Amy Yewdall, Shoupeng Cao, Remco W. van der Hofstad, Samuel Sanchez, Loai K. E. A. Abdelmohsen, Jan C. M. van Hest

**Affiliations:** 1 Department of Bio-Organic Chemistry, Institute of Complex Molecular Systems (ICMS), Eindhoven University of Technology, 5600 MB Eindhoven, The Netherlands; 2 Department of Mathematics and Computer Science, Institute of Complex Molecular Systems (ICMS), Eindhoven University of Technology, 5600 MB Eindhoven, The Netherlands; 3 Institute for Bioengineering of Catalonia (IBEC), The Barcelona Institute of Science and Technology, 08028 Barcelona, Spain; 4 Institució Catalana de Recerca i Estudis Avançats (ICREA), Pg. Lluís Companys 23, 08010 Barcelona, Spain; 5 Present Address: Aragon Institute of Engineering Research (I3A), University of Zaragoza, 50009 Zaragoza, Spain

**Keywords:** Nanocomposites, Molecular machines and motors

## Abstract

Random fluctuations are inherent to all complex molecular systems. Although nature has evolved mechanisms to control stochastic events to achieve the desired biological output, reproducing this in synthetic systems represents a significant challenge. Here we present an artificial platform that enables us to exploit stochasticity to direct motile behavior. We found that enzymes, when confined to the fluidic polymer membrane of a core-shell coacervate, were distributed stochastically in time and space. This resulted in a transient, asymmetric configuration of propulsive units, which imparted motility to such coacervates in presence of substrate. This mechanism was confirmed by stochastic modelling and simulations in silico. Furthermore, we showed that a deeper understanding of the mechanism of stochasticity could be utilized to modulate the motion output. Conceptually, this work represents a leap in design philosophy in the construction of synthetic systems with life-like behaviors.

## Abstract (web summary)

Here the authors develop a coacervate micromotor that can display autonomous motion as a result of stochastic distribution of propelling units. This stochastic-induced mobility is validated and explained through experiments and theory.

## Introduction

It is well recognized that stochastic events play a key directive role in biological processes. In order to provide a robust response to varying input, nature has developed a range of approaches to translate stochasticity into adaptive biological output. Specific examples include the dampening of cellular noise to stabilize stochastic decisions in neuronal cell development^1^ and the autonomous motion of motor proteins^2,3^, a consequence of fluctuating biochemical reactions. Stochasticity is of course not only present in biological systems. Stochastic processes have been observed in many synthetic systems as well and have been used to interpret their specific behavior. For example, in the field of nanomotor and micromotor research, the stochastic positioning of propulsive units has been used as an explanation for the observed motility. Specific examples include the stochastic attachment in a fixed position of enzymes to the surface of nanoparticles by Sanchez et al.^4^ and the conjugation of enzymes to the fluid membrane of liposomes by Sen et al.^5^. However, in contrast to nature, stochasticity has never been used in motor systems, or any other synthetic systems, as a purposely introduced component to control the system’s features. In order to incorporate more life-like behavior in active matter, stochasticity should be given a more prominent role.

Herein we describe a micromotor system in which we have been able to include stochasticity as a design element. Our micromotor is based on a coacervate microdroplet cloaked with a fluidic polymer membrane. To this membrane, enzymes were conjugated as propulsive units. Remarkably, the micromotor did not show the expected motility as a function of enzyme density. This prompted us to develop a physical model and analyze the system in silico. Our model provided us with the insight that a stochastic process governs the motile behavior, which we could subsequently direct, following the model’s guidelines.

## Results and discussion

**Fig. 1:**
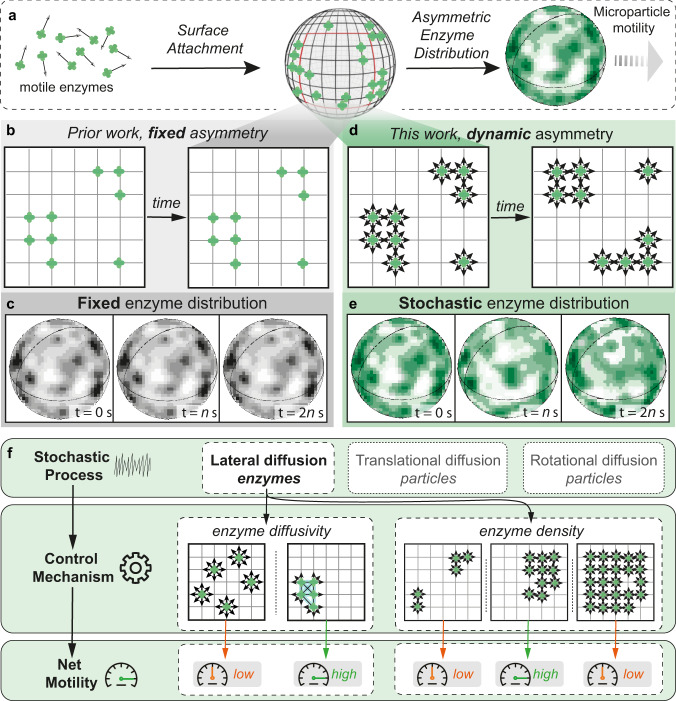
Design philosophy to translate a stochastic process into autonomous motion in an artificial system. **a** Coacervates with surface-confined enzymes and consequent transient asymmetry, leading to autonomous motility of coacervates in the presence of fuel. Schematic that outlines the difference between static (**b**, **c**) and dynamic (**d**, **e**) structural asymmetry. Static asymmetry with fixed enzyme distribution does not change with time (**c**), while dynamic asymmetry features a spatiotemporally stochastic enzyme distribution (**e**). **f** Stochasticity can be utilized for modulating the motion output via tuning either the life time of the transient asymmetry (enzyme diffusivity) or the organizational states of enzyme distribution (enzyme density).

Asymmetry in the motor structure (e.g., shape, catalyst distribution) has been considered as a prerequisite for autonomous motion^6–10^. This has, for example, been achieved by the construction of Janus particles, which are hemi-spherically covered with active catalysts^11–15^. Marangoni flow has also been employed as a propulsion force, e.g., to drive autonomous motion of surfactant-stabilized droplets^16–20^, to induce collective motion^21^, or to navigate in complex environment^22^. Recently, a random surface distribution of enzymes has been shown to propel micromotors in the presence of fuel^4,5^. This inspired us to construct a system, in which asymmetry (i.e., heterogeneous distribution of enzymes) is transient and dynamic, enabled by the inherently fluidic polymer membrane (Fig. 1d, e). This approach is analogous to the work by Sen et al. and in contrast to other investigations that employ static and fixed surface distribution of catalysts (Fig. 1b, c). Our platform was composed of a complex coacervate droplet, which was formed by the spontaneous coacervation of two oppositely charged polyelectrolytes that associate when mixed in water, and which was further stabilized by the presence of a polymer membrane^23^. A more detailed description of the formation process and chemical structures can be found in Supplementary Methods and Supplementary Figs. 1–5. The chemical nature of the membrane made it possible to securely tether enzymes to the surface. Moreover, as this membrane is fluidic at room temperature^24,25^, the continuous spatiotemporal reorganization of such propulsive units was ensured. With this design, we hypothesized that transient asymmetry in enzyme distribution would lead to a variety of random, continuously changing organizational states, with a fraction of these displaying sufficient polarization to generate a net propulsion for coacervates (Fig. 1a).

**Fig. 2:**
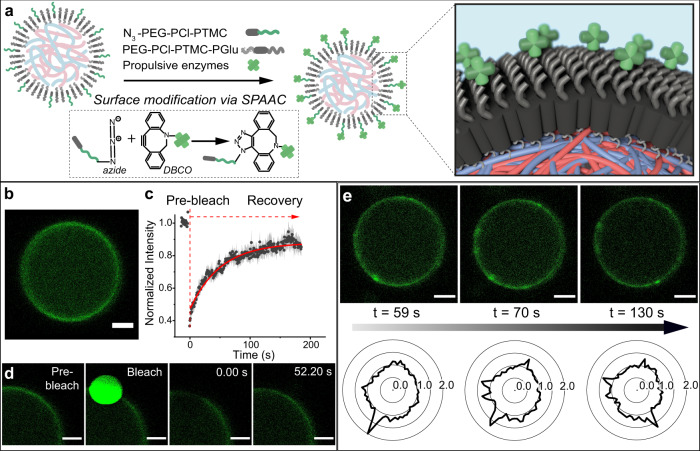
Membrane fluidity resulting in transient asymmetrical distribution of surface-tethered enzymes. **a** Schematic illustration of DBCO-functionalized enzyme attachment on the azido-containing polymeric membrane via strain-promoted alkyne-azide cycloaddition (SPAAC) click reaction. N_3_-PEG-PCL-PTMC represents azide-functionalized polymer and PEG-PCL-PTMC-PGlu represents block terpolymer. **b** Confocal image of a coacervate with successful surface attachment of catalase (mCAT, Cy5 labeled). More than five batches of coacervates show successful enzyme attachment. **c** FRAP recovery curve with black dots representing experimental data (mean ± SEM) and red curve exponential fitting. Three individual batches of coacervates were prepared for FRAP measurements. **d** FRAP measurement showing laser-mediated bleaching of a circular spot on the membrane and subsequent fluorescence recovery of the bleached spot due to Cy5 (green) labeled catalase laterally diffusing along the fluidic membrane. **e** Confocal images taken at different time points (green images) of one single coacervate, surface-functionalized with catalase, with brighter spots along the ring suggesting enzyme clustering. More than three different coacervates were observed to have enzyme clustering. Bottom row displays corresponding intensity radial plots with spikes representing higher local intensity. Radial number of 1.0 indicates evenly distributed enzymes, while radial number >1.0 indicates higher local enzyme density (enzyme clustering). Scale bar 2 µm for **b**, **d**, **e**.

Two enzymes, catalase (CAT) and urease (UR), were selected as the propulsive units due to their reported use as catalytic engines for propelling micron- and nano-sized particles^4,26,27^. The attachment of these enzymes on the coacervate membrane was facilitated by blending in an azide-functionalized polymer, α-azido-poly(ethylene glycol)-*b*-poly(ε-caprolactone-*gradient*-trimethylene carbonate), which rapidly undergoes strain-promoted alkyne-azide cycloaddition with dibenzocyclooctyne-modified enzymes (mCAT and mUR, Fig. 2a). Confocal images confirmed the enzyme-membrane coupling, which was manifested as a ring-like distribution of Cy5-labeled mCAT or mUR (Fig. 2b and Supplementary Figs. 6 and 7). This functionalization strategy retained enzymatic activity (Supplementary Methods) and resulted in membrane-bound enzymes to translocate diffusively along the coacervate surface, as confirmed by fluorescence recovery after photobleaching (FRAP) analysis (Fig. 2d). From the FRAP recovery curve (Fig. 2c), lateral diffusivity of surface-bound mCAT was determined to be 0.035 µm^2^/s (Supplementary Methods). Moreover, the lateral diffusivity of surface-bound mUR was very close to that of mCAT, which was determined to be 0.030 µm^2^/s (Supplementary Fig. 8; for FRAP experimental details, see Supplementary Methods). When the coacervates were analyzed over time, a rare case was observed—a highly punctate, dynamic radial distribution of mCAT was observed (Fig. 2e), confirming our hypothesis that the fluidic polymer membrane results in dynamic enzyme clustering and hence a transient asymmetrical distribution of propulsive units.

**Fig. 3:**
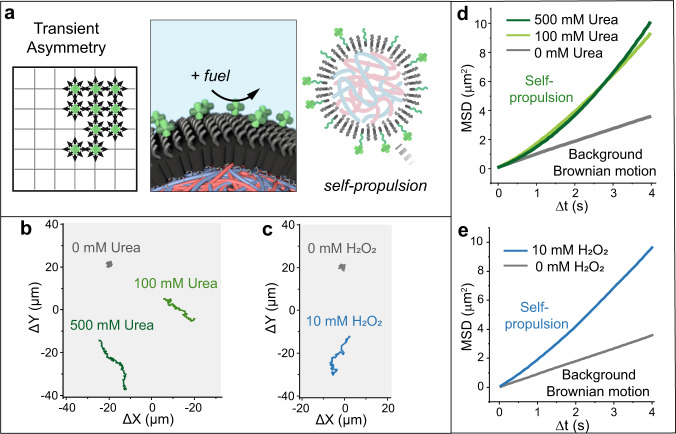
Motility observed in enzyme-tethered coacervates: experimental data. **a** Schematic illustration of transient asymmetry, inducing enzyme-powered self-propulsion. **b** Representative trajectories of mUR-coacervates (diameter ~1.2 µm) over 15 s with 500, 100, and 0 mM urea. **c** Representative trajectories of mCAT-coacervates (diameter ~1.2 µm) with 10 and 0 mM H_2_O_2_, respectively. **d** MSD curves of mUR-coacervates with 500, 100, and 0 mM urea. **e** MSD curves of mCAT-coacervates with 10 and 0 mM H_2_O_2_. MSD curves (**d**, **e**) with error bars (mean ± SEM) are available in Supplementary Fig. 9, *n* = 17 for **d** and *n* = 18 for **e**.

Having confirmed the transient asymmetry on the coacervate membrane, we set out to test coacervate motility. Motion of mCAT- and mUR-coacervates was recorded in the presence or absence of their respective substrate by bright-field microscopy. Videos were recorded at 25 frames per second and for a period of 30–35 s. For each condition, 15–20 coacervates were analyzed. The motile behaviors of both mCAT- and mUR-functionalized coacervates were analyzed by using a tailor-made Python script, the *X* and *Y* trajectory data were extracted, and the mean square displacement (MSD) was calculated (for motility experimental details, see Supplementary Methods). In the absence of fuel, both mCAT- and mUR-coacervates exhibited typical Brownian motion (Supplementary Movies 1 and 2), with linear MSD fitting profiles (Fig. 3d, e and Supplementary Fig. 9). Upon addition of fuel, 10 mM H_2_O_2_ or 500 mM urea (final concentration), respectively, enhanced propulsion with significant increase in MSD profiles was observed for both mCAT- and mUR-coacervates (Supplementary Movies 1 and 2 and Fig. 3d, e). Moreover, individual trajectories (Fig. 3b, c) showed significant path expansion after fuel addition, confirming coacervate propulsion in the presence of fuel, without the need for deliberate structural or externally manipulated asymmetrical features.

**Fig. 4:**
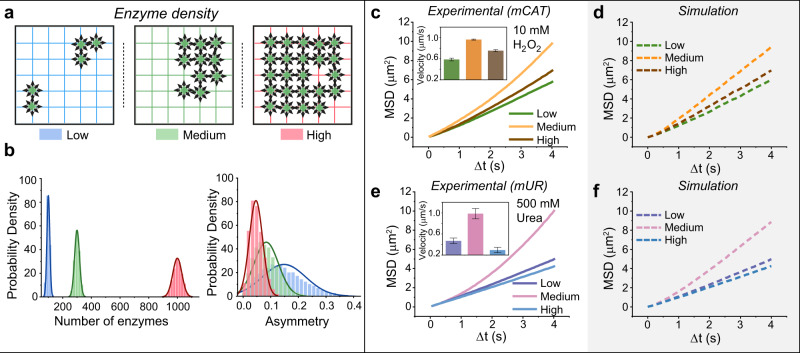
Tuning transient asymmetry by enzyme density varies motion dynamics. **a** Schematic illustration of coacervates with different enzyme densities (low, medium, and high) leading to different organizational states. **b** The distribution of the number of enzymes per coacervate is presented per density based on 100,000 simulated functionalized particles of each density (*λ*_low_ = 100, *λ*_medium_ = 300, *λ*_high_ = 1000). The asymmetry was computed as the net propulsion in the $({{{\boldsymbol{x}}}},{{{\boldsymbol{y}}}})$-plane divided by the number of enzymes (as *v*_c_ = µm/s in these simulations) per coacervate. **c**, **e** MSD curves of mCAT-coacervates/mUR-coacervates (diameter ~ 1.2 µm) with three different enzyme densities, namely, low, medium, and high (Supplementary Table 2). The MSD curves with error bars (mean ± SEM) are available in Supplementary Fig. 10. Inset is the corresponding velocity. Eighteen coacervates were analyzed per condition for mCAT-coacervates and 17 coacervates were analyzed per condition for mUR-coacervates. **d**, **f** MSD curves of mCAT-coacervates/mUR-coacervates predicted by stochastic simulation.

An important parameter that influences the degree of asymmetry in the system, and hence the motile output, is the coverage of enzyme (proportional to number/density of enzymes) on the surface of the coacervates. We set out to test the impact of enzyme density on motility by fabricating both mCAT- and mUR-coacervates with three different surface enzyme densities—low, medium, and high (for exact enzyme number and coverage, see “Methods” and Supplementary Tables 1 and 2). To quantify enzyme density, we first calculated the number of coacervates at a given volume, and then determined, using Cy5-labeled enzymes, the total amount of attached enzymes via fluorescence, as described in the “Methods” section. Obtained enzyme densities and surface coverage of both mCAT- and mUR-coacervates are listed in Supplementary Table 2. Indeed, in the presence of their respective fuels, 10 mM H_2_O_2_ and 500 mM urea, mCAT and mUR-coacervates, with different enzyme densities and same average size (diameter ~ 1.2 µm), moved with different MSDs and velocities (Supplementary Movies 3 and 4). Surprisingly, coacervates with medium enzyme density moved faster than those with high and low densities (Fig. 4c, e and Supplementary Fig. 10), contrary to the monotonic increasing trend one would expect. In addition, the velocity of coacervates with high enzyme density was close to those with low enzyme density. To investigate the mechanism behind the non-monotonic trend of enzyme density and motility, we first ruled out local substrate depletion as the reason for high enzyme density coacervates yielding smaller propulsion. We estimated the relative rate of the enzymatic reaction versus substrate diffusion by calculating the Damköhler number ${{{\rm{Da}}}}=\frac{\dot{r}\,R\,}{d\,{c}_{{{{\rm{sub}}}}}}$. Da of 0.13 and 4.5 × 10^−4^ were obtained for mCAT- and mUR-coacervates, respectively (for details, see “Methods—Estimation of Damköhler number”). In both cases, the Damköhler number is <1 even with the overestimated full coverage of surface with enzymes (experimental enzyme coverage is up to 87%, see Supplementary Table 2). This means that diffusion is faster than the reaction rate and substrate molecules can be resupplied as soon as they react, implying no local substrate depletion.

We therefore hypothesized that the maximal velocity was obtained as a balance between transient asymmetry and enzyme density. The number of enzymes on each coacervate is directly related to the potential of these enzymes to form asymmetric organizational states. For example, a coacervate with maximum enzyme loading will have a saturated, homogeneous distribution of enzymes (Fig. 4a). Hence, in the presence of fuel, such coacervates with on average high symmetry (and thus very low probability of significant asymmetry) will have zero net propulsion. Although at low enzyme density the probability of asymmetric enzyme organization is higher than that at medium enzyme density, the net propulsion is still weaker as fewer enzymes are able to impart velocity, thus yielding a smaller drive capable of actively propelling the coacervate as a whole^4^. In the case of medium enzyme density, there is a favorable balance between the probability of transient asymmetry and the absolute number of enzymes contributing to propulsion, thus resulting in a larger net propulsion than for those with low and high enzyme density.

To demonstrate the interplay of transient asymmetry and enzyme density, a model probability density plot of the degree of asymmetry with different enzyme numbers was derived (Fig. 4b). The measure of asymmetry was defined by the net propulsion divided by the product of the enzyme number and *v*_c_. Then two theoretical extremes were formulated: a uniform (equidistant) organization of enzymes results in 0% asymmetry, while 100% asymmetry is caused by all enzymes clustering at a single point. Sketching the relationship between enzyme density and net propulsion in a schematic illustration shows that the delicate interplay between the parameters is indeed logical (Supplementary Fig. 11) and in agreement with the probability density plot in Fig. 4b.

To further substantiate our hypothesis of transient asymmetry-induced motility, we developed a stochastic mechanical model, which verified our findings from two aspects. First, we parameterized our stochastic model based on the active Brownian particle (ABP) model^28^ to include the mobility and the fluctuations of the enzyme distributions along the surface (see Supplementary Note 1). In this way, we were able to look at the ensemble effect of propulsive units imposed on overall coacervate motion in our system. Second, simulation from the same stochastic model was used to verify the analytical expression and further allowed us to examine the system at the level of the individual enzyme (See Supplementary Note 2).

In general, a stochastic process is a process where an object behaves in a random way. A well-known example is Brownian motion. In our setting, we have three stochastic processes of interest: the inherent translational (1) and rotational (2) diffusion of coacervates on microscale, as well as the lateral diffusivity (3) of the propulsive units (i.e., mCAT or mUR), which is unique to this system. The location of the propulsive units drives the coacervate in a particular direction, governed by the dynamic and asymmetrical distribution of the enzymes on the coacervate surface; the coacervates are reoriented during their motion as a result of the rotational diffusion.

We obtained our stochastic model by modifying the ABP model, which was originally developed to describe the motion of Janus particles^28^. In the ABP model, the catalytic hemisphere defines the direction along which the particle moves, while the rotational diffusion (*τ*_*R*_) randomizes the orientation of the particle making its motion diffusive at long timescales (∆*t* > *τ*_*R*_)^28^. In contrast, the coacervates described in this work do not display a fixed preferential direction since the distribution of the enzyme propulsive units fluctuates with enzymes undergoing lateral diffusion. Our stochastic model assumes that the coacervate moves along the direction of the dipole of the surface distribution with a velocity proportional to its magnitude (see Supplementary Note 1). The MSD after a time ∆*t* was derived as

1 $${{{\rm{MSD}}}}(\Delta t)=\frac{1}{2}{\left(\frac{V}{{D}_{{{{\rm{eff}}}}}}\right)}^{2}({e}^{-2\Delta t{D}_{{{{\rm{eff}}}}}}+2\Delta t{D}_{{{{\rm{eff}}}}}-1)+4{D}_{{{{\rm{T}}}}}\Delta t,$$

where

2 $${D}_{{{{\rm{eff}}}}}={D}_{{{{\rm{R}}}}}+{\left(1+\frac{2}{\lambda }\right)}\frac{{D}_{{{{\rm{L}}}}}}{{R}^{2}},$$

${D}_{{{{\rm{T}}}}}=\frac{{k}_{{{{\rm{B}}}}}T}{6\pi \eta R}$ is the translational diffusion coefficient, *D*_L_ is the lateral diffusion coefficient of the enzymes, ${D}_{{{{\rm{R}}}}}=\frac{{k}_{{{{\rm{B}}}}}T}{8\pi \eta {R}^{3}}$ is the rotational diffusion coefficient of the coacervate, ${k}_{{{{\rm{B}}}}}T$ represents the thermal energy, *η* is the viscosity of the liquid suspending the particle, *R* is the radius of the coacervate, *λ* is the number of enzymes on the coacervate, and *V* is the expected coacervate propelling velocity ($V={v}_{{{{\rm{c}}}}}\sqrt{\frac{2}{3}\lambda }$, where *v*_c_ is the propelling velocity of coacervate induced by one single enzyme).

The (experimental or theoretical) approaches to obtaining these parameters are discussed in the last paragraph of “Methods.” This expression has limiting forms of ${{{\rm{MSD}}}}(\Delta t)=4{D}_{{{{\rm{T}}}}}\Delta t+{V}^{2}\Delta {t}^{2}$ for $\Delta t\ll {\tau }^{\ast }$ and ${{{\rm{MSD}}}}(\Delta t)=(4{D}_{{{{\rm{T}}}}}+{V}^{2}{\tau }^{\ast })\Delta t$ for $\Delta t\gg {\tau }^{\ast }$, where ${\tau }^{\ast }={D}_{{{{\rm{eff}}}}}^{-1}$. The transition between the ballistic (unimpeded motion) and diffusive (random motion resulting from collisions between particles and surrounding molecules) regimes is determined by the new timescale ${\tau }^{\ast }$, which thus depends on both the lateral diffusivity of the enzymes and on the rotational diffusion of the coacervate. It is important to note that enzyme lateral diffusivity contributes to the coacervate velocity and overall MSD in the stochastic model, while the ABP model did not take these two factors into account^28^.

In general, this stochastic model predicts a ballistic motion at short times and a diffusive motion at long times (Supplementary Fig. 12) and this crossover from ballistic to diffusive regimes was indeed observed in the experimental data (Fig. 3d, e and Supplementary Fig. 9). In the case of rapid diffusion of surface-bound enzymes, the lifetime of asymmetry becomes short, which in turn leads to rapid velocity fluctuations. This results in a fast transition of MSD from the ballistic regime to the diffusive regime. Conversely, in the limit of diffusion-limited (static) enzymes, the polarization becomes permanent, leading to recovery of MSD, similar to those of Janus particles (Supplementary Fig. 25)^28^.

To verify the analytically derived MSD and further examine the stochasticity of the system, we studied our system using simulation. The lateral diffusion of a single enzyme on the coacervates’ surface is simulated as a spherical Brownian motion (SBM) with a diffusion coefficient *D*_L_ (see Sections 1 and 3 of Supplementary Note 2 for how such an SBM was simulated)^29^. The fluidity of the coacervates’ membrane leads to stochastic motion of enzymes on the surface, whereas each individual enzyme contributes to the net propulsion of coacervates in the presence of fuel. Important parameters are thus the propelling velocity of the coacervate induced by an individual enzyme (*v*_c_) in the presence of fuel as well as the density of enzymes (*λ*) on the surface; they can both be tuned in our simulation. The direction of the coacervate’s motion is further influenced by its Brownian rotation. Such Brownian rotation is also simulated using an independent SBM with a diffusion coefficient *D*_R_ (see Supplementary Note 2 Section 4). Additionally, the translational diffusion of the coacervate was simulated independently as a two-dimensional Brownian motion. In the case that transient asymmetry highly fluctuates (*D*_L_ >> 1, note: experimental *D*_L_ of mCAT-coacervates is 0.036) or when the rotational diffusion is very fast (*D*_R_ >> 1), the resulting motion of the coacervates was hypothesized to be diffusive (Supplementary Figs. 23–25). The MSDs observed in simulations have a great agreement with the derived analytical expression presented as Eq. (1), as well as with experiments (Supplementary Figs. 12 and 26).

The relationship between enzyme density and net propulsion observed in the experiment (Fig. 4c, e) cannot solely be explained by the change in asymmetry (i.e., $V={v}_{{{{\rm{c}}}}}\sqrt{\frac{2}{3}\lambda }$ is monotonically increasing as a function of *λ*). We thus incorporated enzyme density-dependent specific expected propelling velocities *V* (see Section 5 in Supplementary Note 2) in the stochastic simulation to explain the non-monotonic relation between the MSD and the enzyme density. By altering the number of enzymes in the simulation, MSD curves of low, medium, and high enzyme densities were simulated within the same magnitude of experimental data (Fig. 4d, f). These results demonstrate the potential of harnessing stochastic processes: a seemingly simple, tunable parameter (e.g., enzyme density) can have a profound and non-linear effect on the output behavior (motility).

**Fig. 5:**
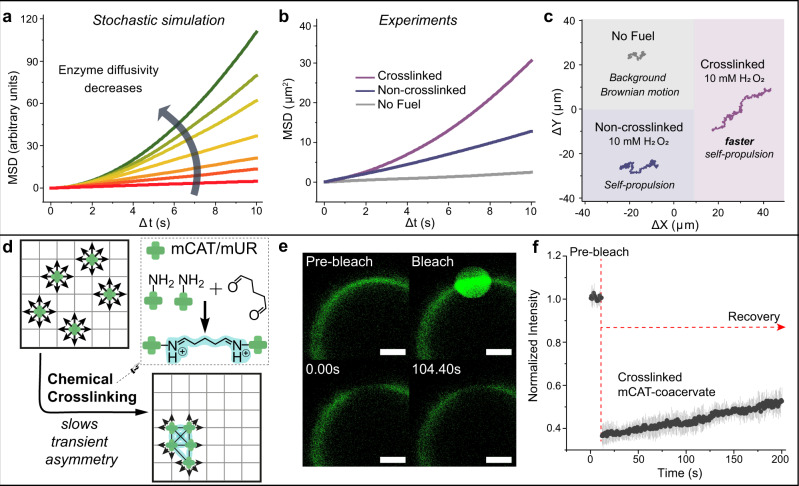
Tuning transient asymmetry by enzyme lateral diffusivity varies motion dynamics. **a** Stochastic simulation predicts that MSD increases with decreasing enzyme diffusivity. **b**, **c** Representative MSD curves and trajectories of non-crosslinked mCAT-coacervates (diameter ~3.6 µm) with no hydrogen peroxide (no fuel), 10 mM peroxide (non-crosslinked), and crosslinked mCAT-coacervates with 10 mM hydrogen peroxide, respectively. MSD curves with error bars (mean ± SEM) are available in Supplementary Fig. 15. **d** Schematic illustration of transient asymmetry slowed down by crosslinking, leading to faster propulsion. **e** FRAP measurements confirmed decreased lateral diffusivity of mCAT after crosslinking, with no significant recovery in the bleached area even 100 s after bleaching (green: Cy5-mCAT). Scale bar represents 2 µm. **f** The fluorescence recovery curve shows only a slight recovery in intensity, indicating that crosslinked mCAT clusters diffuse much more slowly compared to non-crosslinked mCAT. Data are represented as mean ± SEM. Three individual batches of coacervates were prepared for FRAP measurements.

The simulation identified lateral diffusivity of enzymes (*D*_L_) as a key parameter that has a crucial impact on transient asymmetry, due to the speed at which enzyme clusters can form. Therefore, to further control the system with stochastically dominated parameters, we set out to examine the impact of lateral diffusion of enzymes on the motility, by tuning *D*_L_ in the stochastic model. In the extreme case, where *D*_L_ = 0 (completely static motor arrangement), the MSD is at the maximum. While in the theoretical case where *D*_L_ is infinite, dynamic enzyme clustering and its resultant transient asymmetry fluctuate at a rate that is much faster than the measurement timeframe, the MSD profile is the same as that of Brownian motion. Overall, by decreasing *D*_L_ in the stochastic model, an increase in MSDs was predicted (Fig. 5a).

To validate the relationship between transient asymmetry and motility experimentally, we tuned the diffusivity of surface-bound catalase by means of in situ crosslinking of catalase using a chemical crosslinker; namely, glutaraldehyde (Fig. 5). Coacervates with medium mCAT density, the best performer from the enzyme density experiments, were chosen to test this diffusivity hypothesis. In order to avoid aggregation of catalase, crosslinking was carried out after extensive washing steps to remove any unbound catalase (Supplementary Fig. 13). FRAP measurements were performed on mCAT-coacervates with crosslinked surface enzymes to test the extent of decrease in lateral diffusivity. An evident drop in fluorescence recovery speed was observed as there was no significant recovery >100 s after bleaching (Fig. 5e), compared to obvious fluorescence recovery after ~52 s in the case of no crosslinking (Fig. 2d). Although the enzyme diffusivity was drastically diminished, it is important to note that enzymes were not fully static even after crosslinking as a slight recovery in intensity was observed (Fig. 5f).

In order to amplify the motility output, and thus to observe any difference that might arise because of changing the lateral diffusion of enzymes, we aimed at capturing the ballistic regime of MSD curves (rather than the diffusive regime). In order to do so, mCAT-coacervates with average diameter of 3.6 µm (Supplementary Methods and Supplementary Fig. 14) were chosen to perform motility testing, as their characteristic timescale satisfies τ* >> Δ*t*, resulting in a more predominant influence of *D*_L_ on the coacervates’ motion (Eq. (2)). After crosslinking, a clear difference was observed regarding motility: mCAT-coacervates with crosslinked surface enzymes, with the same average size, exhibited increased MSDs and velocities when compared to their non-crosslinked counterparts (Fig. 5b, Supplementary Fig. 15, and Supplementary Movie 5). This propulsive motion can clearly be seen in the movement trajectories, where more stretched and expanded paths were observed for crosslinked ones (Fig. 5c). Should the organizational state of enzymes be permanent and not changing with time, there would not have been a motion enhancement after fixating the enzymes. By tuning enzyme lateral diffusivity, we eventually imposed control over the lifetime of transient asymmetry, which was manifested in the motility output. The enhanced propulsion after crosslinking clearly confirmed the role of the fluidic membrane imparting transient asymmetry and thus motility. Moreover, these experimental results are in line with our prediction by stochastic simulation that lower enzyme diffusivity leads to a more propulsive MSD (Fig. 5a and Supplementary Fig. 23). This agreement between theoretical predictions and experimental validation provides a valuable framework for the incorporation of stochastic mechanisms in synthetic systems.

In addition, an interesting phenomenon was observed that ca. 10% of such non-crosslinked coacervates exhibited anomalous type of motion, so-called run-and-tumble, hitherto only observed in bacteria (e.g., *Escherichia coli*^30^). These non-linear run-and-tumble movements can be characterized by remarkable consecutive alternations between diffusive motion and directional propulsion (Supplementary Movie 6). Analysis of the instantaneous velocity confirmed our observation, with the experimentally observed “run” regime exhibiting significantly higher velocities than predicted by the stochastic model (Supplementary Fig. 31). With our current settings, this run-and-tumble behavior cannot be replicated in the simulation, indicating another degree of stochasticity in the system. As this behavior was only observed with the catalase modified coacervates, we tentatively attribute this phenomenon to a possible explanation—the stochastic release of oxygen microbubbles^31^—but a deeper investigation is needed to substantiate this explanation.

Stochasticity is abundant in nature and of great importance for the regulation of many biological processes. Although this has been well recognized, it has mainly been studied from a phenomenological point of view and its active exploitation is underexplored. This certainly accounts for artificial systems, where researchers have in some cases acknowledged stochasticity to play a key role in the behavior of their systems but have not controlled, let alone designed, this phenomenon. In this contribution, we have demonstrated an engineered motile system that is fully governed by stochasticity. By following a combined experimental and theoretical/simulation approach, we have been able not only to describe the motile process but also to engineer the key parameters that determine stochastic behavior. The great fit between theory and experiment demonstrates that we now have a high level of control over this process. Although our results explain the majority of motile events, we have also observed a small fraction that shows an intriguing “run-and-tumble” behavior. These extreme cases cannot be described by our current model (see Section 6 in Supplementary Note 2) and most probably result from a combination of stochastic processes that we do not fully understand yet. It, however, demonstrates that exploring stochasticity can enable the creation of even more complex, non-linear behavior. For example, research exploring the ability of such stochastic-induced motility to drive chemotaxis is in progress. Hopefully, this study will inspire other researchers to recognize and apply stochasticity as a powerful method to design and engineer other adaptive systems based on fluctuating extrinsic and intrinsic conditions.

## Methods

### Surface enzyme density (mCAT/mUR) of coacervates

To calculate the number of modified enzymes attached on one coacervate, we estimated the number of coacervates per sample and measured the total amount of enzymes attached according to their fluorescence emission.

### Coacervate number estimation

We first measured the density of the interior coacervate phase. The coacervate phase was generated by mixing 6 mL 3 mg/mL Q-Am and 3 mL 3 mg/mL Cm-Am for 5 min (no stabilizing polymer was added). Subsequent centrifugation led to macroscopic phase separation, and supernatant was removed. A total of 10 µL pellet weighed 10.5 µg, therefore the density of the coacervate phase is 1050 mg/mL.

As the mass of Q-Am and Cm-Am and the density of the coacervate phase are known, the total volume of all coacervates was calculated according to ${V}_{{{{\rm{total}}}}}=\frac{{{{{\rm{m}}}}}_{{{{\rm{Q}}}}\mbox{-}{{{\rm{Am}}}}}\,+\,{{{{\rm{m}}}}}_{{{{\rm{Cm}}}}\mbox{-}{{{\rm{Am}}}}}}{{{{\rm{density}}}}}$. The average volume (*V*_avg_) of one coacervate was calculated from the average diameter of the coacervate (obtained from confocal image analysis, see above). By dividing *V*_total_ over *V*_avg_, the number of coacervates was calculated. Results are listed in Supplementary Table 1.

### Amount of mCAT/mUR attached on the coacervates

The amount of mCAT or mUR attached on the coacervates was estimated through Cy5 fluorescence emission (for enzyme modification, see Supplementary Methods). mCAT-coacervates were centrifuged into a pellet, while unbound mCAT stayed in the supernatant. After removing the supernatant, the pellet was resuspended in phosphate-buffered saline buffer. Cy5 fluorescence emission from the supernatant and resuspended pellet were measured by a plate reader (TECAN Spark 10 M). The percentage of mCAT attached was determined by $\frac{{{{\rm{Cy}}}}5\;{{{\rm{emission}}}}\;{{{\rm{in}}}}\;{{{\rm{pellet}}}}}{{{{\rm{Cy}}}}5\;{{{\rm{emission}}}}\;{{{\rm{in}}}}\;{{{\rm{pellet}}}}\;+\;{{{\rm{Cy}}}}5\;{{{\rm{emission}}}}\;{{{\rm{in}}}}\;{{{\rm{supernatant}}}}}$. Then this percentage and the total number of coacervates calculated above were used to calculate the average number of enzyme molecules attached to one coacervate. Results are listed in Supplementary Table 2.

### Estimation of Damköhler number

The relative rate of the enzymatic reaction versus substrate diffusion can be estimated by the Damköhler number:

3 $${{{\rm{Da}}}}=\frac{\dot{r}\,R}{d\,{c}_{{{{\rm{sub}}}}}}$$

where $\dot{r}$ is the reaction rate, *R* is the radius of the coacervate, *d* is the diffusion coefficient of the substrate and *c*_sub_ is the concentration of the substrate far from the coacervate. The reaction rate $\dot{r}$ can be estimated as the product of the maximum turnover rate *k*_cat_ of the enzyme multiplied by the surface density of the enzymes ${{\Gamma } }_{0}$: $\dot{r}\approx {k}_{{{{\rm{cat}}}}}{\Gamma }_{0}$. We estimated surface density of the enzymes in the case of maximum packing (full coverage):

4 $${\Gamma }_{0}\approx \frac{0.91}{\pi {{r}_{{{{\rm{enz}}}}}}^{2}}$$

where *r*_enz_ is the characteristic size of the enzyme. Supplementary Table 3 shows the parameters used for Da, with estimates for mCAT- and mUR-coacervates, respectively.

### Stochastic simulation

The backbone of the stochastic simulation is the simulation of a SBM as described in Section 1 of Supplementary Note 2. The motility of a coacervate with a dynamic membrane was simulated by the union of three independent stochastic processes: (i) the translational diffusion of the coacervate was simulated as a realization of a three-dimensional (3D) Brownian motion depending on the diffusion speed *D*_T_ (see Section 2 of Supplementary Note 2), (ii) the rotational diffusion of the coacervate, causing the direction of the net velocity to change over time, was simulated by implementation of an SBM of one of the unit axes. This way we could implement rotation of the 3D coordinate system at a speed depending on *D*_R_ (see Section 4 of Supplementary Note 2) and (iii) the lateral diffusion of each single enzyme, causing the magnitude of the net velocity to change over time, was simulated as independent SBM at a speed depending on *D*_L_ (see Section 3 of Supplementary Note 2).

### Estimation of simulation parameters

In the stochastic simulation, there were six parameters that influence the motility of enzyme-tethered coacervates, namely, the propelling velocity of coacervates induced by one single enzyme *v*_c_, coacervate radius *R*, translational diffusion coefficient *D*_T_, rotational diffusion coefficient *D*_R_, enzyme lateral diffusion coefficient *D*_L_, and enzyme number on the surface of coacervate *λ*. However, from Eq. (1) it became clear that the impact of *v*_c_ and *λ* could be integrated in the expected coacervate velocity $V={v}_{{{{\rm{c}}}}}\sqrt{\frac{2}{3}\lambda }$ because the influence of *λ* on *D*_eff_ (stated in Eq. (2)) could be ignored for the order of *λ* (shown in Supplementary Table 2). This thus reduces the number of relevant simulation parameters to five.

*R* was obtained from the analysis of coacervate confocal images (Supplementary Fig. 14). To obtain realistic parameter estimates, first $\frac{{k}_{{{{\rm{B}}}}}T}{6\pi \eta }\,$ was estimated to equal 0.14 based on the (experimental) MSD curves of coacervates in the absence of fuel (Supplementary Note 2 Section 2.1) and in turn used to estimate *D*_R_ and *D*_T_ according to the Stokes–Einstein equation. After substituting the estimates of *D*_R_ and *D*_T_ into Eq. (1), this equation was fitted to the experimentally obtained MSD curves to estimate *D*_L_ and *V* for conditions of interest (Supplementary Note 2 Section 5). Besides, the estimate of *V* is within reasonable range of micromotors (see Table in Supplementary Fig. 26) and comparable to other micromotor system, which used the same type of enzyme as propulsion unit^10^.

### Reporting summary

Further information on research design is available in the Nature Research Reporting Summary linked to this article.

## Supplementary information

Supplementary Information

Reporting Summary

## Data Availability

The data that support this study are available from the corresponding author upon reasonable request. Source data are provided with this paper.

## Source data

Source Data

## Code availability

The tailor-made motion tracking Python script is available upon request. The stochastic simulation R script can be accessed on GitHub https://github.com/RAJP93/ETD-of-artificial-cells.git.
