# Author Correction: Engineering transient dynamics of artificial cells by stochastic distribution of enzymes

**DOI:** 10.1038/s41467-021-27682-x

**Published:** 2021-12-16

**Authors:** Shidong Song, Alexander F. Mason, Richard A. J. Post, Marco De Corato, Rafael Mestre, N. Amy Yewdall, Shoupeng Cao, Remco W. van der Hofstad, Samuel Sanchez, Loai K. E. A. Abdelmohsen, Jan C. M. van Hest

**Affiliations:** 1 Department of Bio-Organic Chemistry, Institute of Complex Molecular Systems (ICMS), Eindhoven University of Technology, 5600 MB Eindhoven, The Netherlands; 2 Department of Mathematics and Computer Science, Institute of Complex Molecular Systems (ICMS), Eindhoven University of Technology, 5600 MB Eindhoven, The Netherlands; 3 Institute for Bioengineering of Catalonia (IBEC), The Barcelona Institute of Science and Technology, 08028 Barcelona, Spain; 4 Institució Catalana de Recerca i Estudis Avançats (ICREA), Pg. Lluís Companys 23, 08010 Barcelona, Spain; 5 Present Address: Aragon Institute of Engineering Research (I3A), University of Zaragoza, 50009 Zaragoza, Spain

**Keywords:** Nanocomposites, Molecular machines and motors

Correction to: *Nature Communications* 10.1038/s41467-021-27229-0, published online 25 November 2021.

In this article the affiliation Institució Catalana de Recerca i Estudis Avançats (ICREA), Pg. Lluís Companys 23, 08010 Barcelona, Spain for Samuel Sanchez was missing. The original article has been corrected.

