# Supplementary material for: Engineering transient dynamics of artificial cells by stochastic distribution of enzymes
Source: Nat Commun. 2021 Nov 25;12:6897. doi: 10.1038/s41467-021-27229-0 (PMC8617035; doi:10.1038/s41467-021-27229-0)
Supplement: Supplementary file 1 — Supplementary Information [file 41467_2021_27229_MOESM1_ESM.pdf]

Supplementary Information for

# Engineering transient dynamics of artificial cells by stochastic distribution of enzymes

Shidong Song,† Alexander F. Mason,† Richard A. J. Post,† Marco De Corato, Rafael Mestre, N. Amy Yewdall, Shoupeng Cao, Remco W. van der Hofstad,* Samuel Sanchez,* Loai K. E. A. Abdelmohsen* and Jan C. M. van Hest.*

**This PDF file includes:**

Supplementary Methods
Supplementary Figures 1 to 31
Supplementary Tables 1 to 3
Supplementary Notes 1 and 2

**Other Supplementary Information for this manuscript include the following:**

Supplementary Movies 1 to 6

## Supplementary Methods

### Materials

All materials were used as received unless otherwise stated. For the synthesis of terpolymer: monomethoxy poly(ethylene glycol) 1 and 2 kDa were purchased from Rapp Polymere, trimethylene carbonate was purchased from TCI Europe. For the preparation of modified amylose derivatives: amylose (12-16 kDa) was supplied by Carbosynth and 3-chloro-2-hydroxypropyltrimethyl ammonium chloride (65 wt% in water) was supplied by TCI Europe. Catalase (from bovine liver), urease (Jack bean), dibenzocyclooctyne-PEG4-maleimide (DBCO-PEG4-mal), dibenzocyclooctyne-NHS ester (DBCO-NHS), 30% hydrogen peroxide solution, Atto 488-NHS ester were obtained from Sigma-Aldrich. Sulfo-Cyanine5 NHS ester was obtained from Lumiprobe. 3-Azido-7-hydroxycoumarin was from Baseclick GmbH. Hydrogen peroxide solutions used for motility experiments were prepared by sequential dilutions of 30% hydrogen peroxide solution.

### Synthesis of modified amyloses

Both quaternized (Q-Am) and carboxymethylated (CM-Am) amylose were prepared in accordance with previously published procedures.[1]

Q-Am was prepared by dissolving 1.5 g amylose and 2.78 g NaOH in 14.25 mL Milli-Q at 35 °C. After complete dissolution of the amylose, 11.64 mL 3-chloro-2-hydroxypropyltrimethylammonium chloride solution (60 wt% in water) was added dropwise into the stirring reaction mixture, which was subsequently left to react overnight. After this time, the mixture was neutralized with acetic acid and precipitated into 200 mL cold ethanol. The resulting precipitate was re-dissolved in Milli-Q water and dialyzed extensively against water using regenerated cellulose dialysis tubing (Spectrum Labs, USA) with a 3.5 kDa MWCO before lyophilization. Around 5 g of Q-Am, with a degree of substitution of 0.88, was obtained from this reaction (ca. 80 % yield). $^1$H NMR ($D_2O$) characterization data and chemical structures are presented in Supplementary Fig. 1.

Cm-Am was prepared by dissolving 1.5g amylose and 3.6 g NaOH in 15 mL Milli-Q at 70 °C. After complete dissolution of the amylose, 2.7 g chloroacetic acid was added and the reaction mixture was left to stir for 2 h. After the reaction, the mixture was neutralized with acetic acid and precipitated into 200 mL cold ethanol. The resulting precipitate was re-dissolved in Milli-Q water and dialyzed extensively against water using regenerated cellulose dialysis tubing (Spectrum Labs, USA) with a 3.5 kDa MWCO before lyophilization. Around 5 g Cm-Am, with a degree of substitution of 0.4, was obtained from this reaction (ca. 80 % yield). $^1$H NMR ($D_2O$) characterization data and chemical structures are presented in Supplementary Fig. 1.

## Synthesis of polymers

### Synthesis of poly(ethylene glycol)-b-poly(ε-caprolactone)-g-poly(trimethylene carbonate)-b-poly(glutamic acid) ($PEG_{44}$- b-$PCL_{50}$-g-$PTMC_{50}$-b-$PGA_8$) terpolymer

**Step 1** - Preparation of poly(ethylene glycol)-poly(ε-caprolactone-gradient-trimethylene carbonate) (PEG-PClgTMC). According to a modified literature procedure the organocatalyzed ring-opening polymerization of ε-caprolactone and trimethylene carbonate was performed, aiming for a composition of $PEG_{44}$-$PCL_{50}$-g-$TMC_{50}$ (see reaction scheme in Supplementary Fig. 2).[2] Monomethoxy-PEG-OH macroinitiator (2 kDa, 0.2 mmol, 400 mg) was weighed into a round-bottomed flask along with ε-caprolactone (ε-CL, 10 mmol, 1108 µL) and trimethylene carbonate (TMC, 10 mmol, 1121 mg) and dried via azeotropic evaporation of added toluene (×3). The dried reagents were then re-dissolved in dry toluene (20 mL) and methanesulfonic acid (0.6 mmol, 39 µL) was added, under argon. The reaction mixture was stirred at 30 °C for 6 h, after which time it was precipitated into ice-cold methanol. This yielded 2.1 g of a waxy solid (85 % yield) and the composition of the resulting copolymer was confirmed by $^1$H NMR (Supplementary Fig. 3), comparing the protons of PEG (3.65-3.7 ppm), terminal methyl unit (singlet at 3.40 ppm) to PCL $CH_2$ (multiplet at 2.40-2.25 ppm) and PTMC $CH_2$ (multiplet at 2.2-1.8 ppm). GPC analysis (using a PL gel 5 µm mixed D column, with THF as eluent and PS standards) yielded a Đ of 1.1.

**Step 2** - Chain-end modification with Boc-L-phenylalanine and deprotection. For the incorporation of a terminal amine onto the diblock copolymer, we performed a carbodiimide-mediated esterification between PEG-PCL*g*TMC and Boc-L-phenylalanine (Boc-L-Phe). 1.3 g (ca. 0.1 mmol) of PEG-PCLgTMC copolymer was dissolved in acetonitrile and to it was added *N,N'*-dicyclohexylcarbodiimide (0.2 mmol, 41.9 mg), Boc-L-Phe (0.5 mmol, 23.4 mg), and 4-dimethylaminopyridine (0.02 mmol, 2.5 mg) on ice. The reaction mixture was stirred for 24 h at RT and afterwards placed directly in the freezer overnight to facilitate precipitation of dicyclohexylurea by-product. After cold filtration of the reaction mixture it was concentrated and then precipitated into cold methanol. $^1$H NMR was used to check the product to confirm disappearance of the terminal TMC signal at 1.92 ppm due to addition of phenylalanine at the terminus, and emergence of aromatic protons at around 7.2-7.3 ppm and Boc protons at 1.45 ppm before deprotection. The resulting copolymer was then dissolved in 5 mL DCM, to which 5 mL of trifluoro acetic acid (TFA) was added (on ice) and the mixture was allowed to warm to RT and stirred for 2 h. After 2 h the solvent was evaporated and the copolymer was washed with $NaHCO_3$, 1 M NaCl and brine before drying on $MgSO_4$, filtration and evaporation of the majority of the solvent so that the copolymer could be precipitated into ice-cold methanol. $^1$H NMR clearly showed that the signal arising from the Boc group had disappeared. GPC analysis before and after deprotection yielded a Đ of 1.1, indicating that TFA treatment did not induce copolymer hydrolysis.

**Step 3** - Polymerization and deprotection of *N*-carboxyanhydride γ-benzyl-L-glutamate (NCA-BLG). Following a published method for the controlled polymerization of NCA-BLG by lowering the temperature and maintaining a constant flow of $N_2$ in order to remove $CO_2$ by-product,[3] we aimed to add between 8 and 10 PBLG units onto the amine-terminus of the copolymer

chains. 1 g of Phe-terminated copolymer was weighed into a Schlenk flask and dissolved with ca. 3 mL of dry DMF and cooled in an ice bath. To the cooled mixture 160 mg of NCA-BLG was added under Ar and the reaction was left under a constant flow of $N_2$ for 24 h. The product was precipitated into cold methanol and analysed by $^1$H NMR to confirm the overall composition and, in particular, the presence of benzylic and aromatic protons at 5.0-5.2 and 7.1-7.4 ppm, respectively. Benzyl-protected terpolymer was dissolved in 10 mL THF and 10 mL methanol was added before applying hydrogenation using the H-Cube at 60 °C with 30 bar of $H_2$ pressure and a flow rate of 1 mL min$^{-1}$ to facilitate removal of the benzyl protecting groups. The product was concentrated, then precipitated into cold ether, and dissolved in dioxane before lyophilisation to yield a waxy solid, 0.9 g (85 % yield). $^1$H NMR was used to confirm successful deprotection of the PBLG units, and GPC data indicated that the polydispersity did not increase beyond 1.1 during this process.

**Synthesis of azido-poly(ethylene glycol)-b-poly(ε-caprolactone)-g-poly(trimethylene carbonate) (azido-PEG$_{68}$-b-PCL50-g-PTMC$_{50}$) block copolymers (azido-pol)**

The synthesis protocol was modified from a literature procedure.2 First, azido-PEG-OH macroinitiator (3 kDa, 0.1 mmol, 300 mg) was weighed into a round bottom flask along ε- CL (5 mmol, 553 µL) and TMC (5 mmol, 510 mg) and dried via azeotropic evaporation of added toluene (x3). The dried reagents were then re-dissolved in dry DCM (10 mL) and dry methanesulfonic acid (0.2 mmol, 14 µL) was added under argon. The reaction was stirred at 30 °C overnight, after which there was no sign of unreacted monomers confirmed by 1H NMR. After the reaction was finished, the solution was concentrated and copolymer precipitated into ice cold MeOH (x2), followed by dissolution in dioxane and lyophilization to yield 1.01 g of a waxy solid (78% yield). Copolymer composition was ascertained from the 1H NMR spectrum in the same manner as for step 1 of the terpolymer synthesis above. GPC analysis (using a PL gel 5 µm mixed D column, with THF as eluent and PS standards) yielded Mn = 15.7 kDa, Đ = 1.12. Copolymer composition was confirmed by 1H NMR (Supplementary Fig. 4).

## <u>Enzyme modification and activity test</u>

**Catalase modification: synthesis of mCAT(Cy5-DBCO-Catalase)**

Catalase was modified with Cy5 and DBCO as follows: 20 mg catalase was dissolved in 2.5 mL 0.1 M sodium bicarbonate buffer (adjusted to pH = 8.2) in a glass vial, to which 125 µL of an aqueous solution of sulfo-Cy5-NHS ester (4 mg/mL) was added. This 8:1 (dye: protein) stoichiometry was chosen to ensure sufficient dye labeling as the NHS ester is easily hydrolyzed. The mixture was covered with aluminum foil and stirred overnight at 4°C and then dialyzed for 24 hours against 1x PBS buffer. Cy5 labeled catalase was purified via fast protein liquid chromatography (FPLC, BioRad NGC system). DBCO-PEG4-mal was dissolved in PBS by vigorous vortexing and sonication and added into Cy5-catalase solution. The reaction was carried out overnight by gentle stirring at 4°C. The obtained mCAT was purified by FPLC. The

concentration was determined to be 2.3 mg/mL by Nanodrop. Cy5 labelling number per catalase tetramer was determined to be 2 by UV-vis spectroscopy. The presence of DBCO on catalase was confirmed by fluorescence emission after addition of the fluorogenic agent 3-azido-7-hydroxycoumarin. 3-azido-7-hydroxycoumarin is highly quenched and upon azido-alkyne reaction with DBCO, the fluorescence is restored.

**Urease modification: synthesis of mUR(Cy5-DBCO-Urease)**

Urease was modified with Sulfo-Cy5-NHS ester and DBCO-NHS ester as follows: 100 mg urease was dissolved in 10 mL 0.1 M sodium bicarbonate buffer (adjusted to pH = 8.2) in a glass vial, to which 1.14 mg sulfo-Cy5-NHS ester powder and 0.3 mg DBCO-NHS ester (in DMSO)were added. The excess of NHS ester was to ensure sufficient dye labeling as NHS ester is easily hydrolyzed. The mixture was covered with aluminum foil and stirred overnight at 4°C and then dialyzed for 24 hours against 1x PBS buffer. The obtained mUR was then purified with FPLC. The concentration was determined to be 3.7 mg/mL by Nanodrop. Cy5 labelling number per urease was determined to be 1.4 by UV-Vis spectroscopy. The presence of DBCO on mUR was confirmed by fluorescence emission after addition of the fluorogenic agent 3-azido-7-hydroxycoumarin.

**Bovine serum albumin modification**

Bovine serum albumin (BSA) was succinylated and modified with dye Atto 488 as follows: 5 mg BSA was dissolved in 2.5 mL 100 mM $NaHCO_3$ (adjusted to pH 9) in a 1.5 mL Eppendorf tube (BSA concentration 2.5 mg/mL), to which 20 µL Atto 488 -NHS ester (10 mM, in DMSO) was subsequently added. The reaction mixture was gently stirred at room temperature overnight. The next day, succinic anhydride was added directly to the reaction mixture at a final concentration of 50 mM. The reaction mixture was gently stirred under aluminum foil for another 4 hours before purification by FPLC and concentration to approximately 1.2 mg/mL. Atto 488 labeling number per BSA was determined to be 1.2 by UV-Vis spectroscopy.

**Catalase activity assay before and after modification**

The activity of mCAT was determined by measuring the decomposition of hydrogen peroxide over time via UV-Vis spectroscopy (JASCO V-650). In these assays, 0.1 mL of diluted catalase (unmodified or modified) in PBS solution was mixed with 2.9 mL hydrogen peroxide solution (0.036 wt% in water), and the characteristic absorbance at 240 nm ($A_{240}$) was monitored over time. Enzyme activity was calculated according to:

$$\text{Units/mL enzyme} = \frac{3.45 \times \text{df}}{\text{time} \times 0.1},$$

where 3.45 corresponds to the decomposition of 3.45 µmoles of hydrogen peroxide in a 3.0 ml reaction mixture producing a decrease in the $A_{240}$ from 0.45 to 0.40, df is the dilution factor and time (minutes is the time for $A_{240}$ to decrease from 0.45 to 0.40. Enzyme activities of unmodified

catalase and modified catalase were determined after 3 individual measurements to be about $10\times10^3$ U/mg and $5\times10^3$ U/mg respectively.

**Urease activity assay before and after modification**

Urease activity was tested by measuring pH change in solution upon addition of its substrate urea. First the pH of urease solution was adjusted to around 4.0 by HCl addition, then urea was added and pH change over time was recorded. Ten minutes after urea addition, the pH recording stopped. Urease activity before and after modification were both tested. After modification, urease kept about 74% activity according to:

$$\text{Relative activity} = \frac{\Delta\text{pH (urease after modification)}}{\Delta\text{pH (urease before modification)}}.$$

**Enzyme activity test after surface attachment via SPAAC reaction**

Catalase activity was tested after surface attachment qualitatively. After surface attachment and removal of unbound catalase via centrifugation, 0.2 mL 5 wt% $H_2O_2$ was added to 0.2 mL mCAT-coacervate suspension in PBS buffer, and the generation of a large amount of oxygen bubbles confirmed catalase was still active after surface attachment.

## Fabrication of mCAT or mUR functionalized coacervates

Coacervates were prepared based on a sonication method as follows: to 200 μL 0.5 mg/mL quaternized amylose (Q-Am) solution in an Eppendorf tube was added 100 μL 0.5 mg/mL carboxymethyl amylose (Cm-Am) solution to induce coacervation. The mixture was placed in a sonication bath for 2 minutes to allow the coacervate growth, followed by an addition of 10 μL 50 mg/mL polymer mixture (20%v/v 50 mg/mL azido-$PEG_{68}$-*b*-$PCL_{50}$-*g*-$PTMC_{50}$ and 80%v/v 50 mg/mL terpolymer in PEG 350) to generate a polymeric membrane and stabilize the coacervate core. The size of such coacervates was effectively limited to 1-3 μm by utilizing sonication during coacervate droplet growth. Coacervates larger than 3 μm could also be generated by using a method based on employing an Eppendorf shaker as previously reported.[1] Large coacervates were prepared *via* the sonication method stated above, except the amylose mixture was placed in an Eppendorf shaker instead of a sonication bath. A broad range of coacervate sizes was achieved through the combination of changing mixing methods (shaker or sonication), altering volume and growth time of the coacervate core. Supplementary Figure 14 shows the size distribution of coacervates with diameters of 1.2 ± 0.4 μm, 2.6 ± 0.7 μm, 3.6 ± 1.6 μm and 10.3 ± 3.4 μm.

After the assembly of coacervates, certain amounts (listed below) of mCAT (Cy5-DBCO-Catalase) or mUR (Cy5-DBCO-Urease) were added to allow mCAT / mUR attachment on the polymeric membrane through strain-promoted azide–alkyne cycloaddition (SPAAC) between the azido-group installed on the polymer membrane and the DBCO moiety introduced in the enzyme.

The reaction was carried out for two hours, followed by separation of unbound enzymes by centrifugation and refreshing the supernatant with buffer. Surface enzyme density (Fig. 4) was tuned by changing the added amount of mCAT or mUR. For mCAT-coacervates, low, medium and high surface density corresponded to 4.6, 46, or 81 μg mCAT addition. For mUR-coacervates, low, medium and high surface density corresponded to 5.4, 54, or 108 μg mUR addition. The actual density of both enzymes on the surface of coacervates was calculated using fluorescence spectroscopy (vide infra).

## Size analysis of coacervates

The size of the coacervates was obtained by confocal image analysis. To facilitate size analysis, succinylated Atto 488 modified BSA (sBSA) was loaded in the interior of the coacervates. To assemble sBSA loaded coacervates, after addition of Cm-Am, 10 μL sBSA was added to the amylose mixture, followed by a growth time of 2 min and addition of the membrane-forming terpolymer.

Confocal laser scanning microscopy (Leica TCS SP8) was used to capture images of sBSA-loaded coacervates with a 488nm laser line using a 63×, 1.20 NA water immersion objective. The pinhole was set to 1 Airy Unit (156 μm). The images were then converted to binary, and the area of each coacervate was analyzed using software ImageJ. The diameter of coacervates was obtained by averaging at least 100 coacervates.

## Motility test for mCAT-/mUR-coacervates

### Experimental chamber

A simple experimental chamber was designed and prepared to minimize side effects that could be mistaken as self-propulsion, such as drift or solution evaporation. This chamber was made from two glass microscopy slides spaced by two pieces of autoclave tape. Autoclave tape was first attached to a larger glass slide on two ends, followed by the addition of sample in the middle, and capping by a smaller glass slide.

### Optical recording

The videos of coacervate motion were recorded using a camera (Hamamatsu Digital Camera C11440) on an inverted optical microscope (Leica DMi8). A 63× water immersion objective was used for this recording. The coacervate suspension and their corresponding substrate (hydrogen peroxide or urea) were first mixed thoroughly and immediately added to the chamber. The recording started shortly after. For mCAT-coacervates, hydrogen peroxide concentration after mixing was set to 10 mM. This very low concentration was chosen to avoid oxygen bubble generation that could disturb the liquid inside the chamber and mask self-propulsion. Sample in the experimental chamber was replaced at least every 3 min to maintain the same substrate concentration.

**Data analysis of motion**

A tailor-made Python script was used to track the coacervates and obtain the mean square displacement (MSD). MSD is a measure of deviation of the position of a particle with respect to its initial position over time. It is commonly used to analyze the dynamics of self-propelled particles and is calculated as below in 2D projection:

$$\mathrm{MSD}\ (t) = \langle(\vec{x}(t) - \vec{x}\ (0))^2\rangle,$$

where $\vec{x}(0)$ is the initial position of the coacervate, and $\vec{x}(t)$ is the position of the coacervate when time is *t*. The obtained MSD was plotted against the time interval *Δt*. To extract the velocity of the particles in the self-propulsive regime, a least-squares fitting to the equation $\mathrm{MSD}(\Delta t) = 4D_T\Delta t + V^2\Delta t^2$ was performed using OriginLab software.

## In situ crosslinking of mCAT on the polymeric membrane

After mCAT coacervate fabrication, 109 µL of glutaraldehyde dissolved in 1x PBS (GA, 275 mM) and 91 µL of 1x PBS were added to 100 µL mCAT coacervate suspension. The final concentration of GA was 100 mM. The reaction was carried out for 3 hours at room temperature with mild stirring. Unreacted GA was removed by centrifugation and resuspension twice.

## Fluorescence recovery after photobleaching of enzyme-attached coacervate membrane

To monitor the lateral diffusion of surface-attached enzymes (mCAT and mUR), fluorescence recovery after photobleaching (FRAP) (Leica TCS SP8 confocal microscope) was performed on the membrane. A selected circular area on the membrane was bleached (100% laser intensity), and the fluorescence intensity in the bleached area was monitored (1-2% laser intensity) over time. Faster fluorescence recovery in the bleached area indicated faster lateral diffusion of enzymes on the membrane. As micron-sized particles have a characteristic rotational time (the time required for a particle to rotate 360° in solution around its axis) due to Brownian motion, we chose coacervates with diameters of 10 µm (with a characteristic rotational time of ~500 s) for the FRAP measurements (substantial recovery observed after 52 s in Fig. 2D) to minimize the effect of rotation on apparent lateral diffusivity.

Data was analyzed as follows: Fluorescence intensity at time *t*, *I*(*t*) was background subtracted and corrected for unintentional photobleaching, which was calculated as

$$I(t) = \frac{\mathrm{ROI}(t) - \mathrm{Bg}}{\mathrm{Ref}(t) - \mathrm{Bg}},$$

where ROI*(t)* is the average intensity of the bleached area at time *t*, Ref*(t)* is the average intensity of an unbleached fluorescent area (same size as bleached area) and Bg is the average intensity of a background area. *I*(*t*) was further normalized such that pre-bleach intensity was set to 1. This was done by dividing *I*(*t*) by average background-subtracted pre-bleach intensity within the bleach area.

Normalized intensity $I(t)_{normalized}$ was plotted against time to get the recovery curve. This recovery curve was fitted with $I(t)_{normalized} = B - Ae^{-\tau t}$, assuming exponential kinetics, to obtain parameter $\tau$. In the above equation, $\tau$ is the recovery time constant and $A$ and $B$ are two constants. Then recovery half-time $\tau_{1/2}$ and apparent diffusion coefficient $D_L$ were calculated from $\tau_{1/2} = -ln0.5 / \tau$ and $D_L = 0.88\ \omega^2 / 2\tau_{1/2}$ (for a circular bleaching area) respectively.[4] We therefore obtained a $D_L$ of 0.0352 ± 0.0008 $\mu m^2$ / s for mCAT-coacervates and a $D_L$ of 0.0298 ± 0.0005 $\mu m^2$ / s for mUR-coacervates. The results were obtained by measuring at least 6 different enzyme-attached coacervates for both cases.

FRAP was performed on crosslinked mCAT-coacervates following the same protocol. The recovery curve was plotted in the same way, however, the fitting equation for non-crosslinked mCAT coacervate was not suitable for the crosslinked coacervates because the interaction between catalase enzymes (because of crosslinking) complicated the fluorescence recovery, which was not taken into account in the fitting equation.[5]

## Supplementary Figures

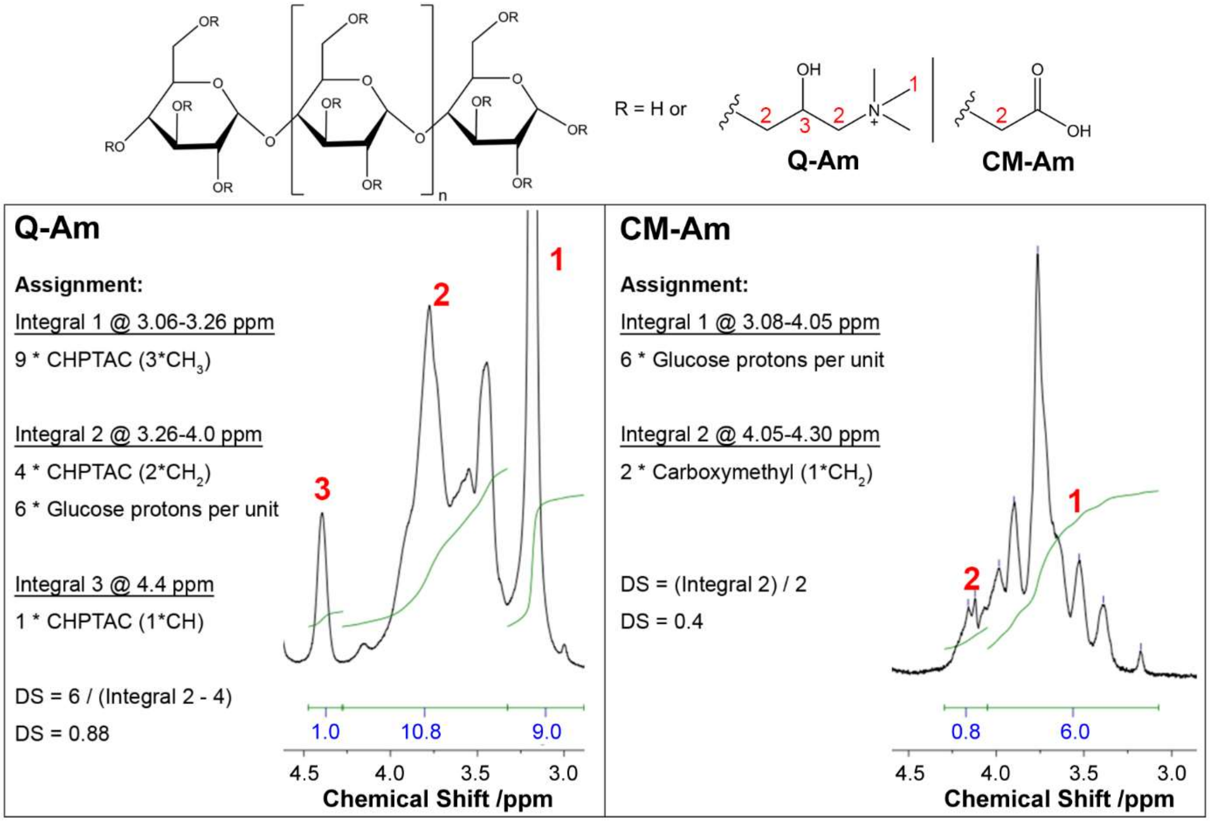


## Figure 1.

$^{1}$H NMR processing of highly charged amylose derivatives, including calculation of the degree of substitution (DS = number of modifying groups per glucose unit), of both quaternized (Q-Am) and carboxymethylated (Cm-Am) products.

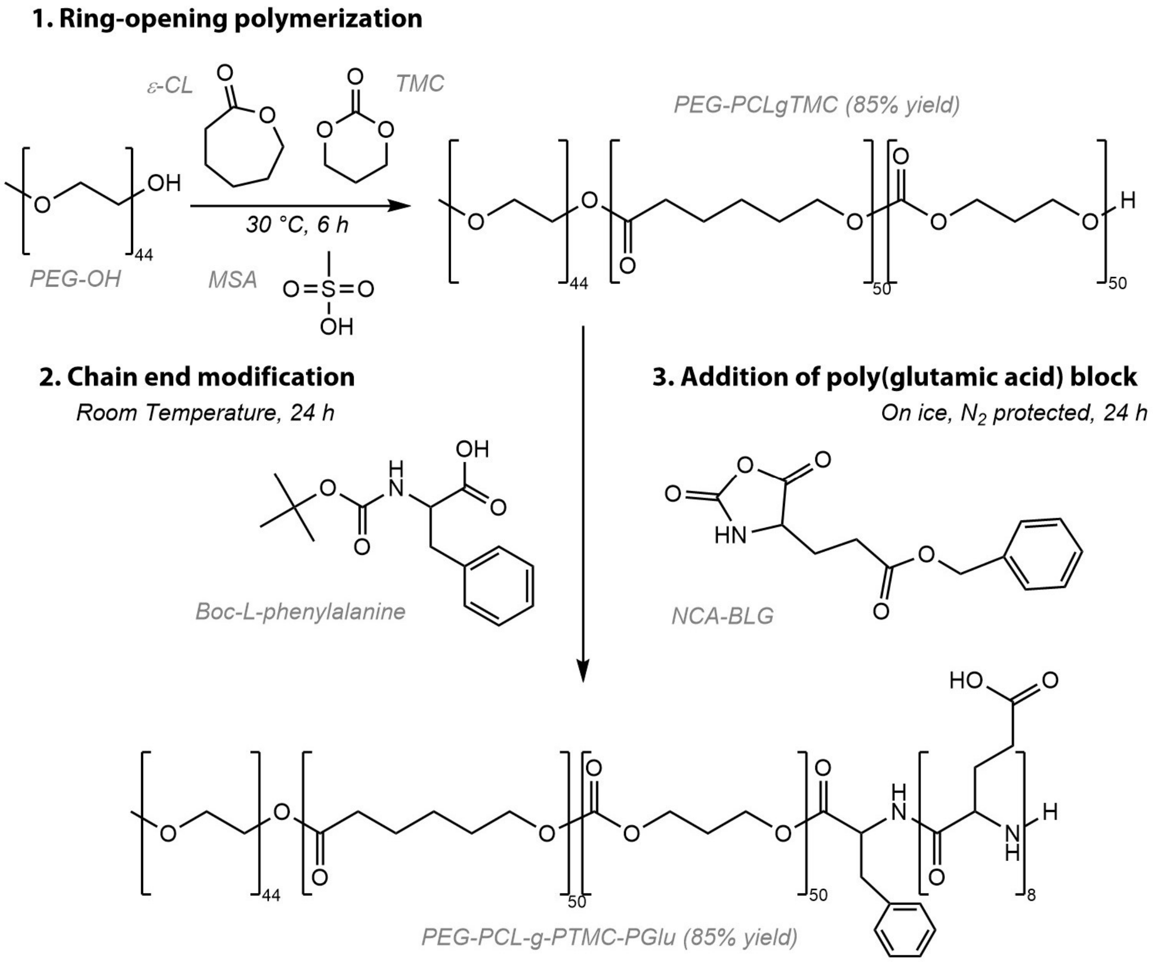


## Figure 2.

Synthesis of $PEG_{44}$-b-$PCL_{50}$-g-$PTMC_{50}$-b-$PGA_8$ terpolymer. Poly(ethylene glycol)$_{44}$ monomethyl ether was used to initiate the ring opening polymerisation of ε-caprolactone and trimethylene carbonate (step 1). The terminal alcohol of this polymer was subsequently modified via a Steglich esterification with Boc-L-Phe-OH to yield a primary amine after TFA deprotection (step 2). The final poly(L-glutamic acid) block was introduced by the ring opening polymerization of *N*-carboxyanhydride γ-benzyl-L-glutamate, followed by hydrogenation (step 3).

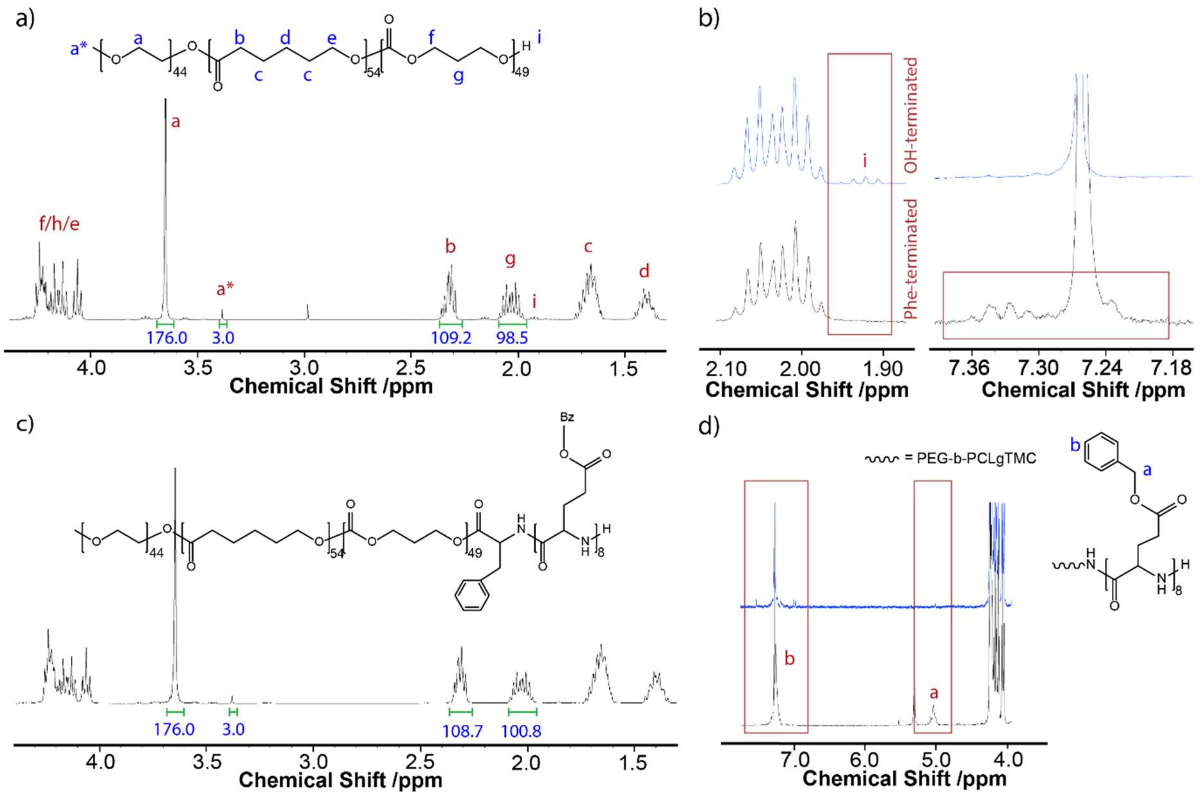


**Figure 3.**

$^{1}$H NMR processing of terpolymer synthesis. (a) analysis of PEG-PCLgPTMC, (b) demonstration of the complete chain-end modification of PEG-PCLgPTMC with Boc-L-Phe, (c) analysis of the product of ring opening polymerization of BLG-NCA and (d) specific evaluation of PBLG content of the resulting terpolymer using benzylic and aromatic protons at 5.0-5.2 and 7.1-7.4 ppm, with upper spectrum showing successful deprotection by hydrogenation.

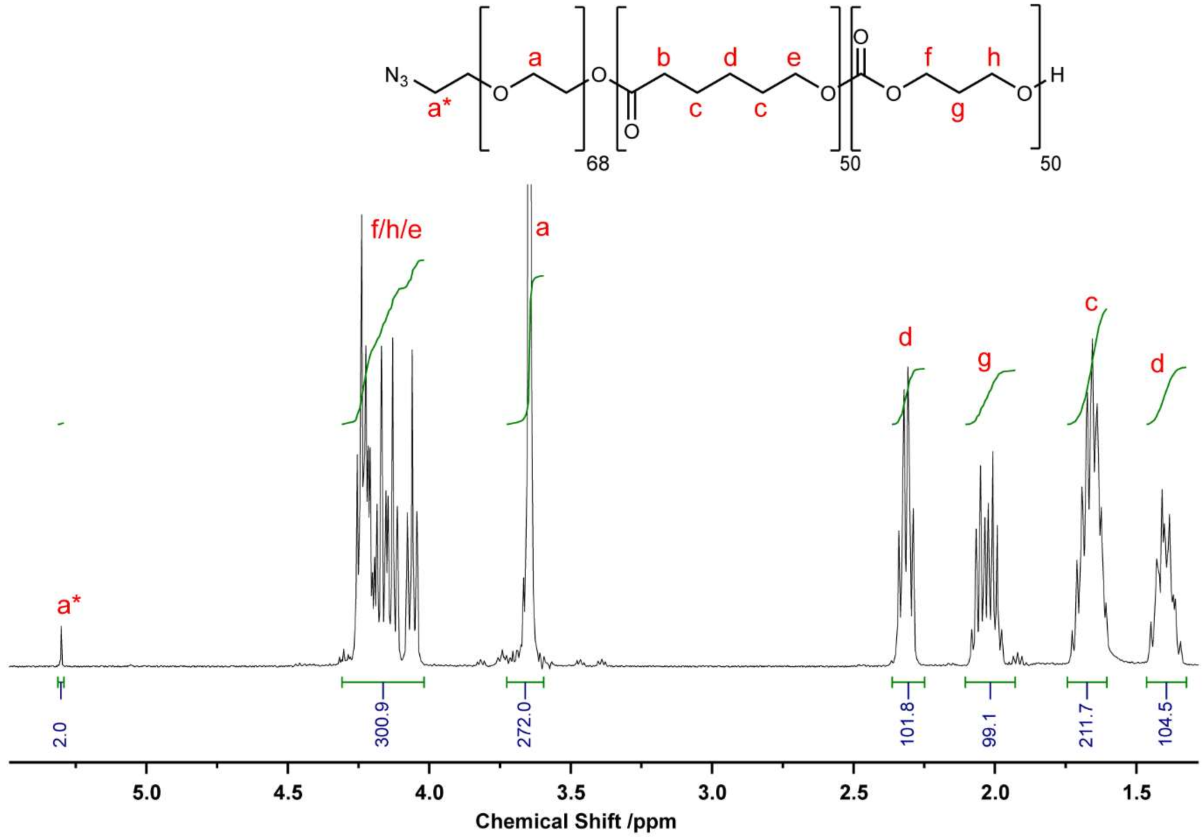


**Figure 4.**

$^{1}$H NMR processing of azido-PEG$_{68}$-*b*-PCL$_{50}$-*g*-PTMC$_{50}$. This spectrum confirms $M_n$ by comparing the integrals of the PEG block to the new PCL and PTMC blocks, and the retention of azido functionality (a*).

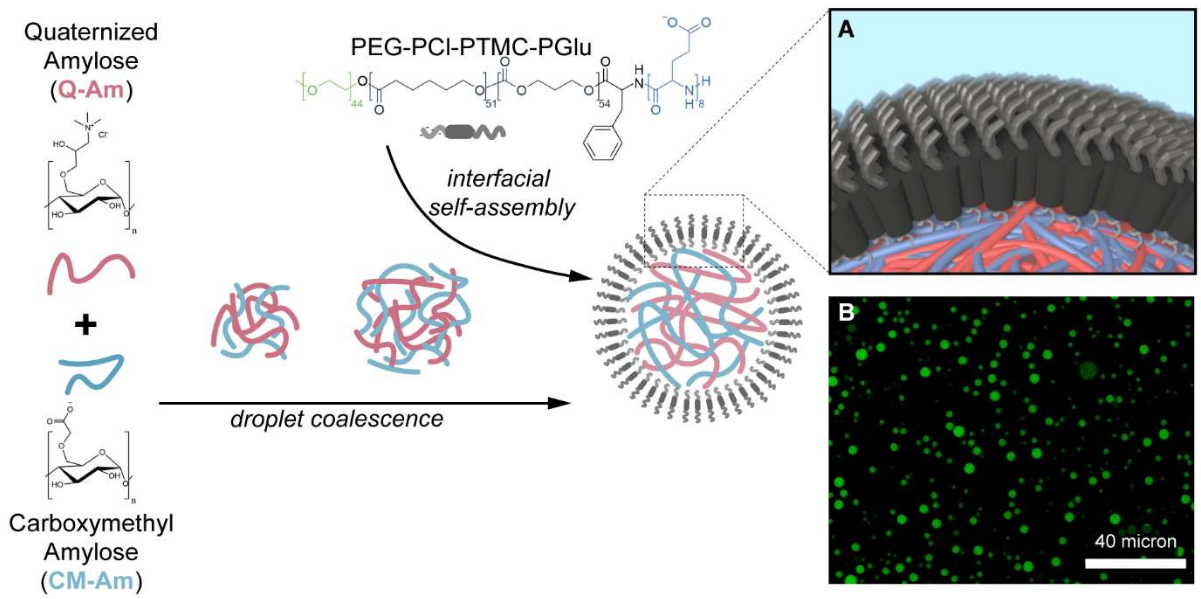


**Figure 5.**

The formation of terpolymer stabilized coacervates proceeds via hierarchical self-assembly. First, oppositely-charged modified amylose polymers are mixed, immediately forming complex coacervate microdroplets that coalesce and increase in size over time. This growth is arrested by the addition of a carefully-designed block terpolymer, PEG-PCL-PTMC-PGlu, which self-assembles on the surface of coacervate microdroplets (A), driven by electrostatic attraction between PGlu (negative) and Q-Am (positive). Large, stable populations (B) of coacervates are thus obtained.

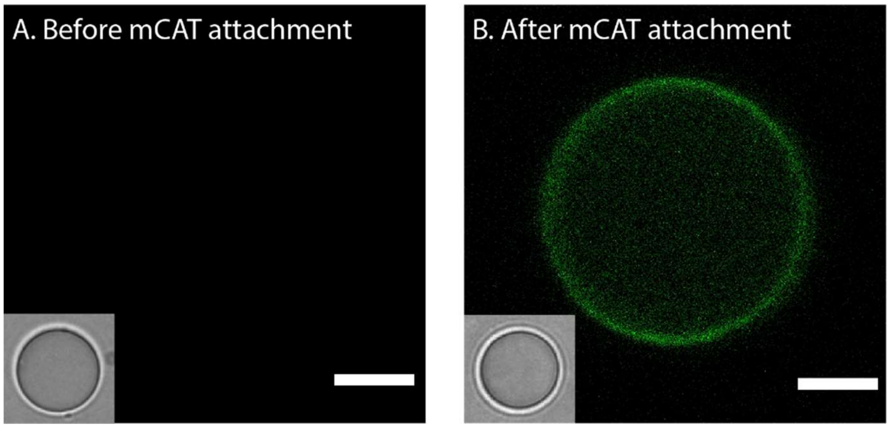


**Figure 6.**

Before (A) and after (B) mCAT attachment on the surface of coacervates. A ring structure was seen in (B) indicating surface-dominated attachment of enzyme. Inserts are corresponding Bright-field images. Scale bar represents 2 μm. More than 5 batches of coacervates show successful enzyme attachment.

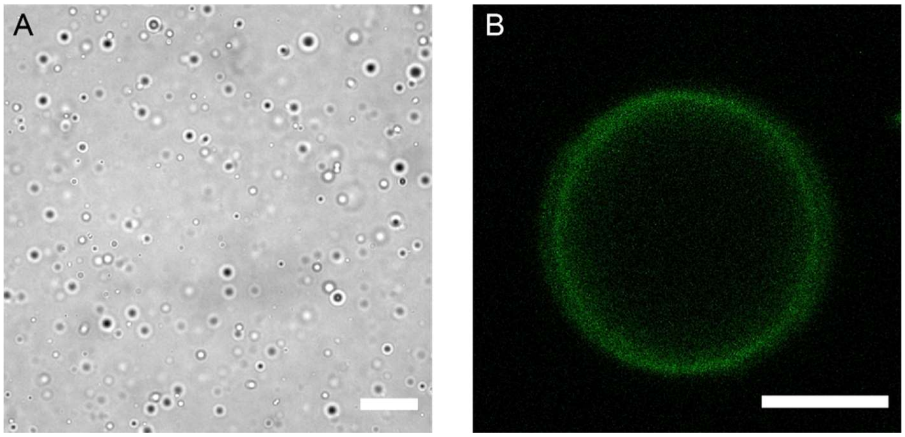


**Figure 7.**

Bright field (A) and confocal image (B) of mUR-coacervate. The ring-like structure in (B) confirmed the successful enzyme-membrane coupling. Scale bar represents 20 µm and 5 µm. More than 5 batches of coacervates show successful enzyme attachment.

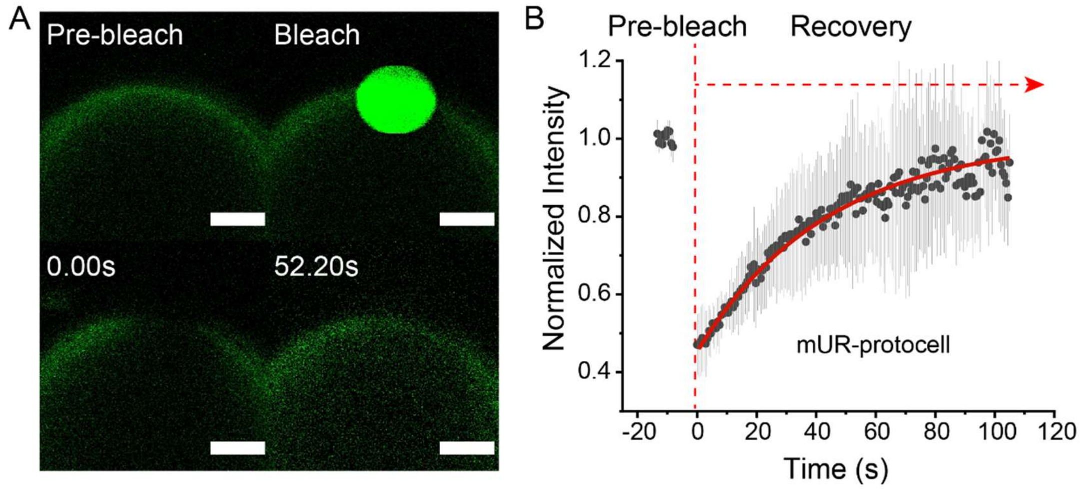


**Figure 8.**

FRAP measurements were performed on mUR-coacervates to test the lateral diffusivity of urease. (A) Substantial recovery 52.2 seconds after laser bleaching was observed (Green: Cy5 from mCAT). (B) FRAP recovery curve with black dots representing experimental data (n = 5, mean ± SEM) and red curve exponential fitting. Lateral diffusivity of surface-bound mUR was determined to be around 0.03 $\mu m^2/s$. Scale bar represents 2 μm. Three individual batches of coacervates were prepared for FRAP measurements.

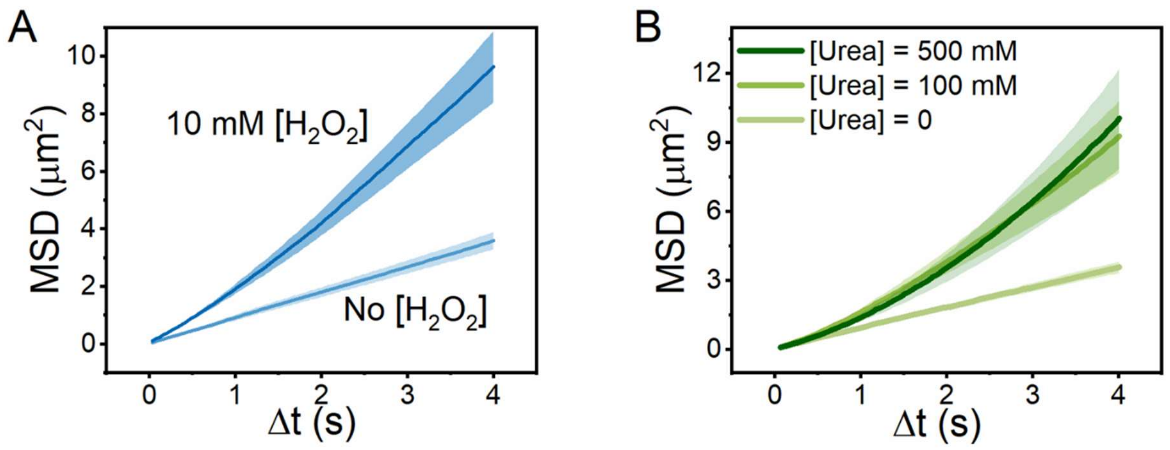


**Figure 9.**
MSD curves of mCAT-coacervates (A) (n = 18, mean ± SEM) and mUR-coacervates (B) (n = 17, mean ± SEM) in the absence / presence of their respective fuels.

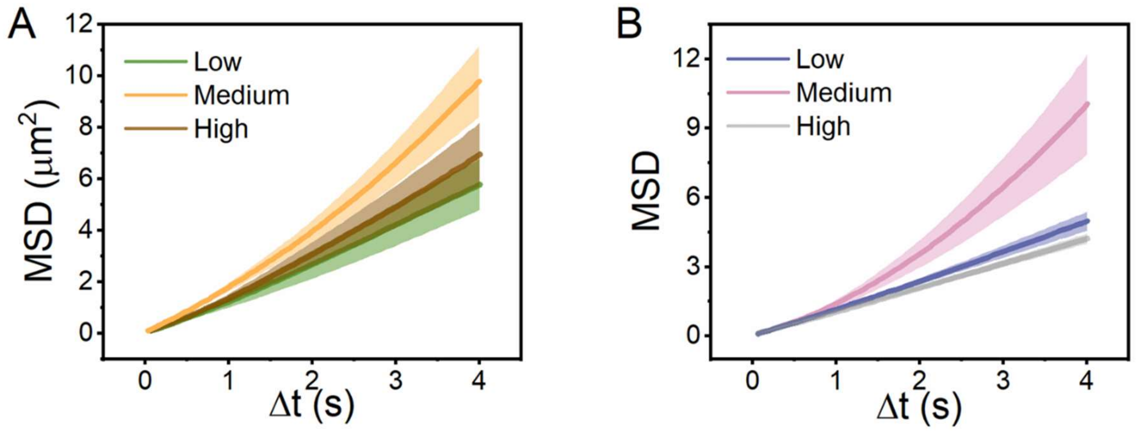


**Figure 10.**

MSD curves of mCAT-coacervates (A) and mUR-coacervates (B) with three different enzyme densities, namely low, medium, high. (A) Hydrogen peroxide concentration = 10 mM (n = 18, mean ± SEM). (B) Urea concentration = 500 mM (n = 17, mean ± SEM).

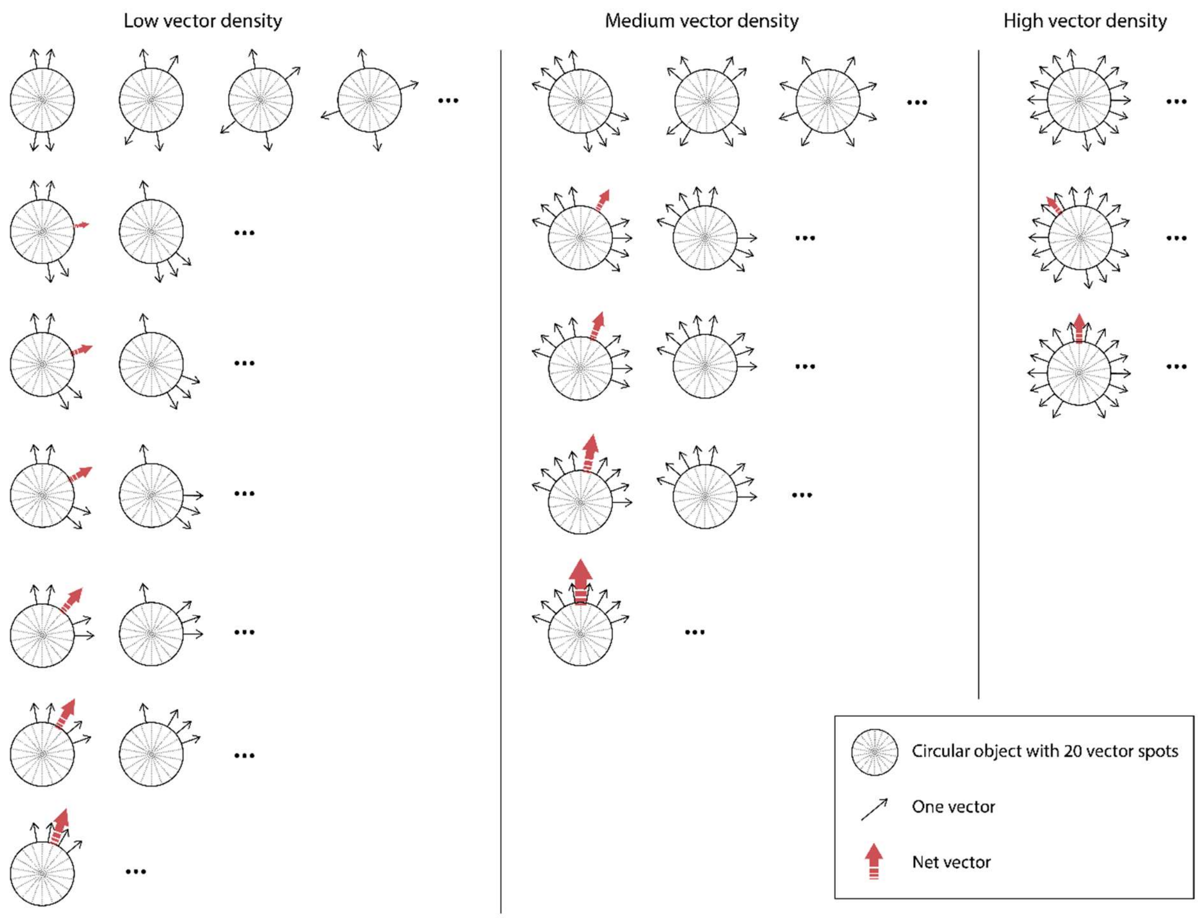


**Figure 11.**

Schematic illustration of relationship between vector density (enzyme density in 2D) and total generated propulsion. Please note: this figure is only for illustrating the idea of interplay, and it doesn't imply the moving direction of enzymes or coacervates.

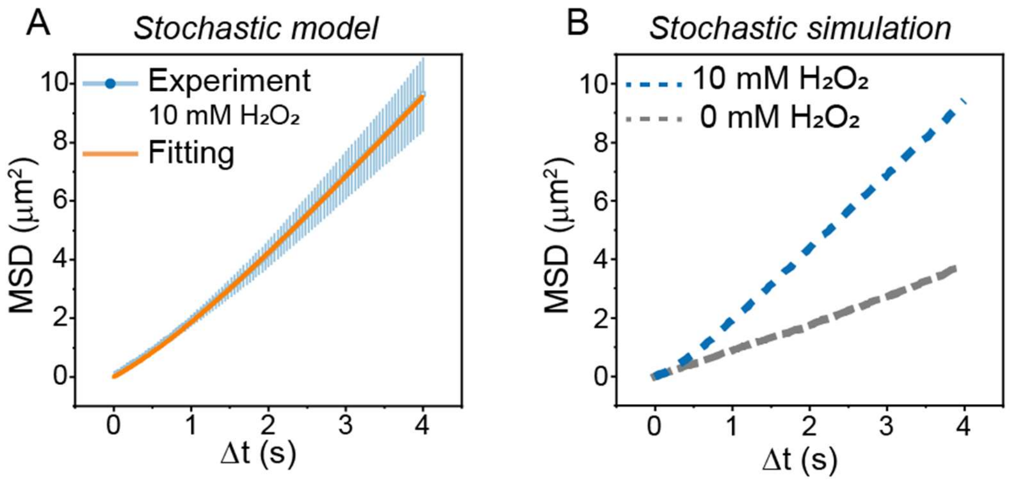


# Figure 12.

(A) Fitting of MSD curve of mCAT-coacervate with 10 mM $H_2O_2$ with stochastic model. (B) The predicted MSD curves of mCAT-coacervate with 10 mM and 0 mM $H_2O_2$ with stochastic simulation.

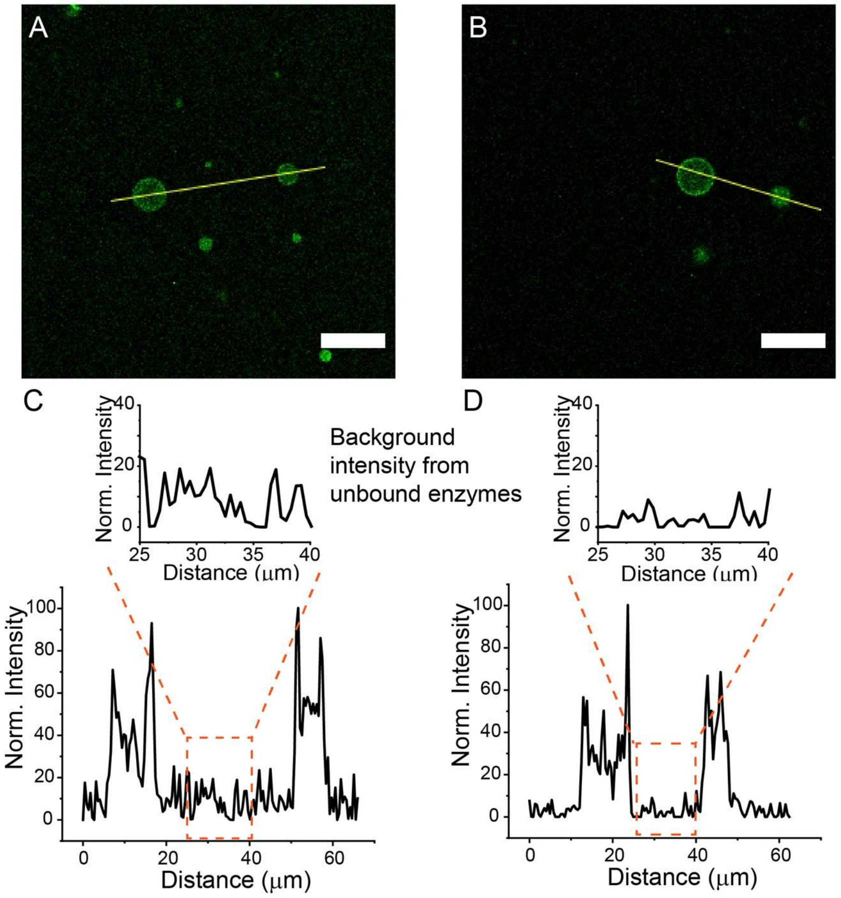


**Figure 13.**

mCAT-coacervates before (A) and after (B) unbound mCAT removal by centrifugation and resuspension. Line intensity profiles of corresponding yellow lines in (A) and (B) show the background fluorescence intensity after unbound mCAT removal (D) is significantly lower than before (C), indicating the effectiveness of said purification. More than three individual batches of coacervates were observed to have similar results. Scale bar represents 20 μm.

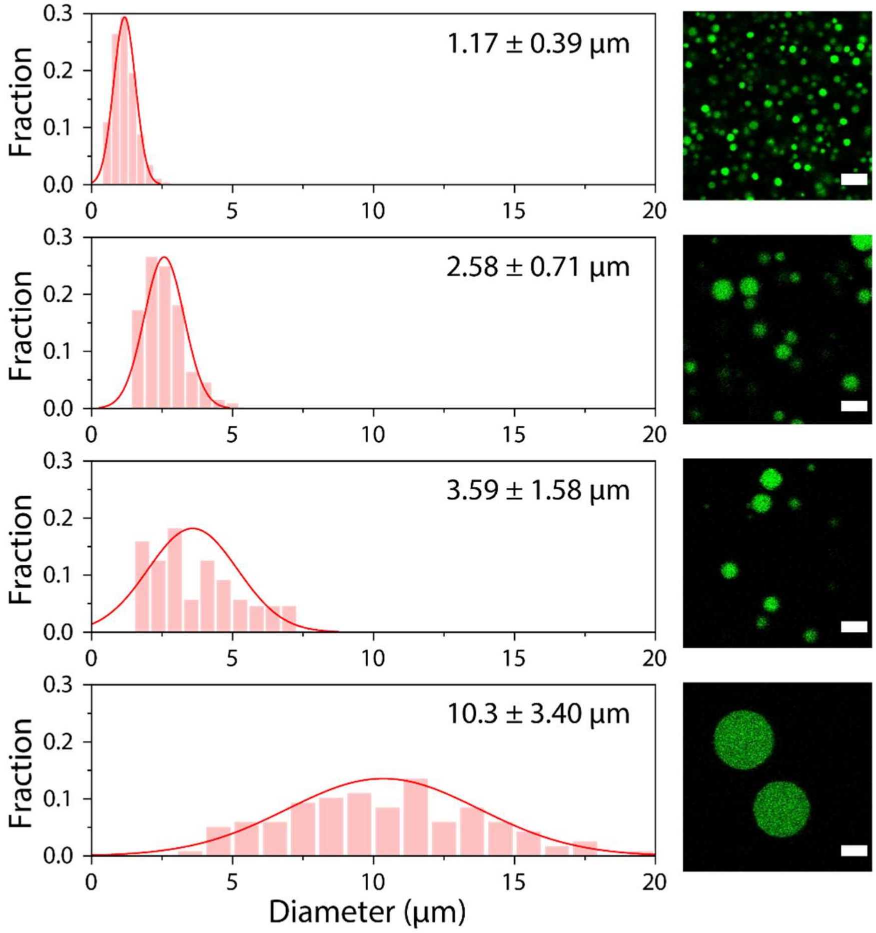


**Figure 14.**

The size of coacervates can be finely tuned by adjusting amylose derivative volume and altering the preparation method (shaker or sonication). Coacervates with diameter of 1.17 ± 0.39 µm were prepared by the sonication method with growth time of 1.5 min; coacervates of 2.58 ± 0.71 µm were prepared by the shaker method with 0.2 unit volume (40 µL Q-Am + 20 µL Cm-Am); coacervates of 3.59 ± 1.58 µm were prepared by the shaker method with 0.4 unit volume (100 µL Q-Am + 50 µL Cm-Am); coacervates of 10.3 ± 3.40 µm were prepared by the shaker method with 1 unit volume (200 µL Q-Am + 100 µL Cm-Am). For all cases, n > 100. Three individual batches of coacervates were prepared for size measurements. Scale bar represents 5 µm.

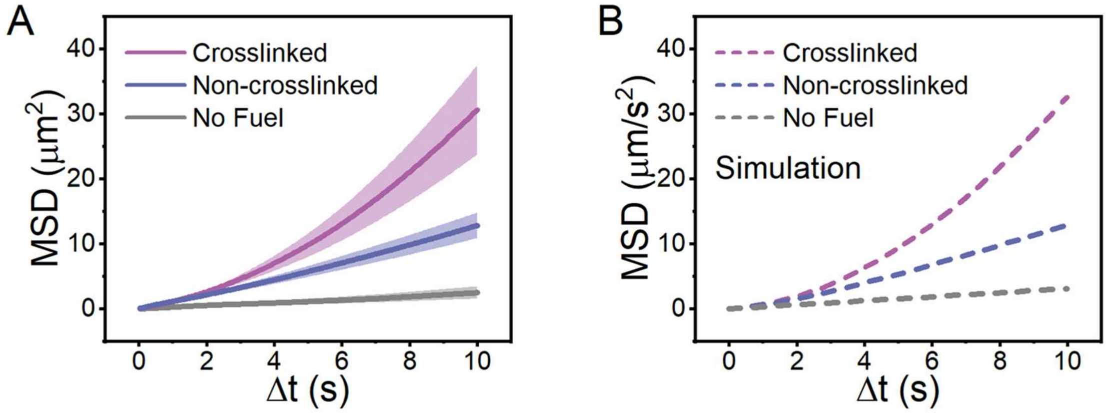


**Figure 15.**

(A) MSD curves of non-crosslinked mCAT-coacervates (diameter~3.6 μm) with no hydrogen peroxide (no fuel), 10 mM peroxide (non-crosslinked) and crosslinked mCAT-coacervates with 10 mM peroxide. For all conditions, n = 18, and data are represented as mean ± SEM. (B) MSD curves predicted by stochastic simulation.

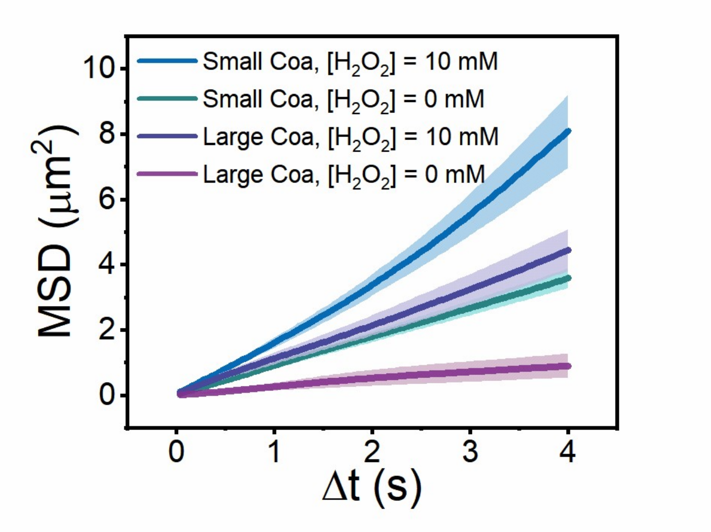


**Figure 16.**

MSD curves of mCAT-coacervates with different sizes. Small Coa represents coacervates with diameter of 1.2 ± 0.4 μm and Large Coa represents coacervates with diameter of 3.6 ± 1.6 μm. For all conditions, n = 18, and data are represented as mean ± SEM.

## Supplementary tables

### Table 1.

Coacervate number per mL with different sizes.

| Diameter of coacervates (μm) | Number of coacervates / mL |
|---|---|
| 10.3 | $800 \times 10^3$ |
| 3.6 | $29 \times 10^6$ |
| 1.2 | $510 \times 10^6$ |

### Table 2.

Number density and surface area coverage of mCAT/mUR attached on one coacervate.

| mUR-coacervate | | | mCAT-coacervate | | |
|---|---|---|---|---|---|
| Average diameter /μm | 1.2 ± 0.4 | | Average diameter /μm | 1.2 ± 0.4 | |
| Enzyme density | Number of mUR /coacervate | Coverage of enzyme /% | Enzyme density | Number of mCAT /coacervate | Coverage of enzyme /% |
| Low | $5 \times 10^3 – 7 \times 10^3$ | 2 - 3 | Low | $6 \times 10^3 – 8 \times 10^3$ | 2 - 3 |
| Medium | $40 \times 10^3 – 50 \times 10^3$ | 17 - 22 | Medium | $100 \times 10^3 – 120 \times 10^3$ | 43 - 52 |
| High | $100 \times 10^3 – 120 \times 10^3$ | 43 - 52 | High | $150 \times 10^3 – 200 \times 10^3$ | 65 - 87 |

**Table 3.**

Parameters used for estimation of Damköhler number.

| | mCAT-coacervate | mUR-coacervate |
|---|---|---|
| $R$ (μm) | 0.60 | 0.60 |
| Substrate | Hydrogen peroxide | Urea |
| $d$ ($\times 10^{-9}$ $m^2/s$) | 0.90* | 1.30* |
| $c_{sub}$ (mM) | 10 | 100 |
| $c_{sub}$ (number of molecule/$m^3$) | $6.022 \times 10^{24}$ | $6.022 \times 10^{25}$ |
| $r_{enz}$ (nm) | 5* | 7* |
| $k_{cat}$ ($s^{-1}$) | $1.00 \times 10^5$ | $1.00 \times 10^4$ |
| Da | 0.13 | 0.00045 |

*Data sources: Diffusion coefficient of hydrogen peroxide and urea,[6] hydrodynamic radius of catalase[7] and urease.[8]

# Supplementary Note 1: Stochastic model by modifying Active Brownian Particle model

We consider a spherical particle of radius $R$. Enzymes are attached on the surface of the sphere and can diffuse along the surface with lateral diffusion coefficient $D_L$ . Due to the Brownian motion of the enzymes at the surface, the distribution of enzymes fluctuates around the homogeneous equilibrium concentration $\Gamma_0$.

We assume that the particle velocity, is proportional to the instantaneous dipolar distribution of the enzymes:

$$\mathbf{V}(t) = \lambda v_c\left(p_z(t)\mathbf{e}_z + p_x(t)\mathbf{e}_x + p_y(t)\mathbf{e}_y\right) = \lambda v_c \mathbf{p}(t). \tag{1}$$

The proportionality constant $v_c$ is a characteristic velocity scale **per enzyme** that depends on the specific phoretic propulsion mechanism, $\lambda$ represents the total number of enzymes and the unit vectors $\boldsymbol{e}_i$, with $i = x, y, z$, define the reference frame of the particle and $p_i$, with $i = x, y, z$, define the components of the dipole. The dimensionless vector $\mathbf{p}(t)$ defines the instantaneous direction and magnitude of the dipolar distribution relative to the equilibrium concentration $\Gamma_0$.

The dynamics of the particle position vector are given by the superposition of the self-propulsive velocity and the translational Brownian motion:

$$\frac{d\mathbf{r}(t)}{dt} = \lambda v_c \mathbf{p}(t) + \boldsymbol{\xi}(t). \tag{2}$$

With the translational noise due to Brownian motion given by a Gaussian random variable with zero mean and delta correlated in time $\langle \boldsymbol{\xi}(t)\boldsymbol{\xi}(t') \rangle = 2D_t \mathbf{I}\, \delta(t - t')$, with $D_T$ the translational diffusion coefficient of the particle. This model is reminiscent of the Active Brownian Particle (ABP) model, with the difference that the dipole $\mathbf{p}(t)$ changes due to the surface diffusion of the enzymes and due to the rigid-body rotations of the particle.

To close the problem, we need to evaluate the evolution of the dipolar concentration of the enzyme $\mathbf{p}(t)$. To do so we derive a Langevin equation for the vector $\mathbf{p}(t)$.

## 1 Langevin equation for the fluctuating dipole

We assume that the fluctuations of the enzyme concentration are small and can be computed as if the system was in equilibrium.[9] Considering the full non-equilibrium features of the fluctuations would couple different spatiotemporal modes and different fields, which would make the problem extremely complicated. We believe that these interesting aspects deserve a separate analysis.

Using the framework of linear fluctuating hydrodynamics, it can be shown that the dipolar concentration vector, scaled by equilibrium distribution $\Gamma_0$, obeys the Langevin equation

$$\frac{d\mathbf{p}(t)}{dt} = -\frac{2D_L}{R^2}\mathbf{p}(t) - \boldsymbol{\xi}_{dip}(t) + \boldsymbol{\xi}_{rot}(t), \tag{3}$$

where the noise $\boldsymbol{\xi}_{rot}(t)$ represents the change of dipole orientation due to the rigid-body rotational Brownian motion of the particle. This noise is Gaussian, it has zero average and it is delta-correlated in time [10,11]

$$\langle \boldsymbol{\xi}_{rot}(t)\boldsymbol{\xi}_{rot}(t')\rangle = 2D_R\left(\mathbf{I} - \frac{\mathbf{p}(t)\mathbf{p}(t)}{\mathbf{p}(t)\cdot\mathbf{p}(t)}\right)\delta(t-t'), \tag{4}$$

where $D_R$ is the rotational diffusion coefficient of the particle, which is given by the Stokes-Einstein formula

$$D_R = \frac{k_B T}{8\pi\eta R^3} \tag{5}$$

where $k_B T$ represents the thermal energy and $\eta$ is the viscosity of the liquid suspending the particle The surface diffusion of the enzymes contributes to the fluctuations of the dipole through the term $\boldsymbol{\xi}_{dip}(t)$, which is Gaussian-distributed with zero mean and delta-correlated in time

$$\langle \boldsymbol{\xi}_{dip}(t)\boldsymbol{\xi}_{dip}(t')\rangle = \frac{4\,D_L}{R^2\lambda}\mathbf{I}\delta(t-t'). \tag{6}$$

The first term on the right-hand side describes the tendency of entropy to restore a homogeneous distribution of enzymes.

We can decompose the equation for the dipolar vector into an equation for its magnitude and an equation for its orientation that is expressed as the unit vector $\widehat{\mathbf{p}}(t) = \frac{\mathbf{p}(t)}{\|\mathbf{p}(t)\|}$. To obtain an equation for the unit vector defining the orientation of the dipole, $\widehat{\mathbf{p}}(t)$, we multiply the equation above by the tensor $\mathbf{I} - \widehat{\mathbf{p}}(t)\widehat{\mathbf{p}}(t)$

$$\frac{d\widehat{\mathbf{p}}(t)}{dt} = \left(\boldsymbol{\xi}_{rot}(t) - \boldsymbol{\xi}_{dip}(t)\right)\cdot\left(\mathbf{I} - \widehat{\mathbf{p}}(t)\widehat{\mathbf{p}}(t)\right). \tag{7}$$

The symmetric tensor $\mathbf{I} - \widehat{\mathbf{p}}(t)\widehat{\mathbf{p}}(t)$ selects the components of the noise that are perpendicular to $\mathbf{p}(t)$, which do not change its norm. Above we have used the fact that $\frac{d\mathbf{p}(t)}{dt}\cdot\left(\mathbf{I} - \widehat{\mathbf{p}}(t)\widehat{\mathbf{p}}(t)\right) = \frac{d\widehat{\mathbf{p}}(t)}{dt}$. By using the property that $(\mathbf{I} - \widehat{\mathbf{p}}(t)\widehat{\mathbf{p}}(t))^2 = \mathbf{I} - \widehat{\mathbf{p}}(t)\widehat{\mathbf{p}}(t)$ and the fact that the rotational noise, $\boldsymbol{\xi}_{rot}(t)$, is by definition perpendicular to $\mathbf{p}(t)$, we include the projection tensor directly in the definition of the noise, and rewrite the above equation as

$$\frac{d\widehat{\mathbf{p}}(t)}{dt} = \boldsymbol{\xi}_{eff}(t). \tag{8}$$

With the noise having variance given by

$$\langle \boldsymbol{\xi}_{eff}(t)\boldsymbol{\xi}_{eff}(t')\rangle = 2\left(\frac{2D_L}{R^2\lambda} + D_R\right)\left(\mathbf{I} - \widehat{\mathbf{p}}(t)\widehat{\mathbf{p}}(t)\right)\delta(t-t'). \tag{9}$$

The above equation states that the dipole orientation fluctuations are due to the sum of the rigid body rotation of the sphere and the surface diffusion of the enzymes. The characteristic time associated to the change of orientation of the instantaneous dipole is given by

$$\tau_{eff} = \left(\frac{2\,D_L}{R^2\lambda} + D_R\right)^{-1}. \tag{10}$$

The correlation of the orientation at different times is therefore given by[10]

$$\langle \widehat{\mathbf{p}}(t)\cdot\widehat{\mathbf{p}}(t')\rangle = e^{-2|t-t'|/\tau_{eff}}. \tag{11}$$

To obtain an equation for the modulus of $\mathbf{p}(t)$ of the dipole, we multiply the first equation with $\widehat{\mathbf{p}}(t)$ to obtain

$$\frac{dp(t)}{dt} = -\frac{2D_L}{R^2} p(t) - \boldsymbol{\xi}_{dip}(t) \cdot \hat{\mathbf{p}}(t), \tag{12}$$

where we denoted the modulus of $\mathbf{p}(t)$ as $p(t)$ and we used the fact that $\frac{d\mathbf{p}(t)}{dt} \cdot \hat{\mathbf{p}}(t) = \frac{dp(t)}{dt}$.

The noise is a scalar and its variance can be shown to be given by

$$\langle \boldsymbol{\xi}_{dip}(t) \cdot \hat{\mathbf{p}}(t)\, \boldsymbol{\xi}_{dip}(t') \cdot \hat{\mathbf{p}}(t') \rangle = \frac{4\, D_L}{R^2 \lambda} \delta(t - t'). \tag{13}$$

The above equation states that the modulus of the dipolar enzymatic concentration is given by an Ornstein-Uhlenbeck process.[11] Straightforward integration of the equation yields the correlation[11]

$$\langle p(t) p(t') \rangle = \frac{1}{\lambda} e^{-2|t-t'|/\tau_L}, \tag{14}$$

with the characteristic time $\tau_L = \frac{R^2}{D_L}$. The same-time magnitude variance is inversely proportional to the total number of enzymes at the surface $\lambda$.

Finally, it can be shown that the modulus and the orientation of the dipole are two uncorrelated random processes.

## 2 Evaluation of MSD

We rewrite the equation of motion of the particle as

$$\frac{d\mathbf{r}(t)}{dt} = \lambda v_c p(t) \hat{\mathbf{p}}(t) + \boldsymbol{\xi}(t). \tag{15}$$

The solution of the equation is given by

$$\mathbf{r}(t) = \lambda v_c \int_0^t p(s)\, \hat{\mathbf{p}}(s)\, ds + \int_0^t d\mathbf{W}(s) + \mathbf{r}(0). \tag{16}$$

With $d\mathbf{W}(s)$ is an increment of a Wiener process with zero mean and variance $\langle d\mathbf{W}(s) d\mathbf{W}(s') \rangle = 2D_T \mathbf{I}\, \delta(t - t')$.

The mean square displacement (MSD) of the particle is calculated as

$$\langle (\mathbf{r}(t) - \mathbf{r}(0))^{\mathbf{2}} \rangle = \lambda^2 v_c^2 \int_0^t \int_0^t \langle p(s)\, \hat{\mathbf{p}}(s) \cdot p(s')\, \hat{\mathbf{p}}(s') \rangle\, ds\, ds'$$
$$+ \int_0^t \int_0^t \langle d\mathbf{W}(s) \cdot d\mathbf{W}(s') \rangle ds\, ds'. \tag{17}$$

By plugging in the definition of the correlations of $p(t)$, $\hat{\mathbf{p}}(t)$ and of the Wiener process $d\mathbf{W}(s)$ we obtain

$$\langle (\mathbf{r}(t) - \mathbf{r}(0))^{\mathbf{2}} \rangle = \frac{\lambda\, v_c^2}{2\, D_{eff}^2} \left[ e^{-2\, t\, D_{eff}} - 1 + 2\, t\, D_{eff} \right] + 6 D_T t. \tag{18}$$

The MSD has the same functional form as that found in the case of the Active Brownian Particle (ABP) model.[12] Note that this relation is equivalent to Equation (1) in the main text and Equation (14) in Supplementary Note 2. We find a ballistic regime at small times and a diffusive regime at long times. The main difference between the MSD predicted by the ABP and that predicted by the equation above is given by the characteristic timescale over which the particle

transitions from the ballistic regime to the diffusive regime, $\tau^* = {D_{eff}}^{-1}$, is set by the inverse an effective rotational diffusion coefficient, given by

$$D_{eff} = D_R + \left(1 + \frac{2}{\lambda}\right)\frac{D_L}{R^2}. \tag{19}$$

While for the ABP model this characteristic timescale is simple given by $\frac{1}{D_R}$. Compared to the ABP model, $\tau^*$ depends on the surface diffusion coefficient and on the total number of adsorbed enzymes $\lambda$. Usually, there are many enzymes adsorbed on the surface of the particle and $\frac{2}{\lambda} \ll 1$.

In the case of mobile enzymes, their surface diffusion contributes to the change of the dipole orientation, which determines the direction of the self-propulsion. This means that there are two mechanisms responsible for the change of the dipolar distribution of enzymes: (i) diffusion of the enzymes along the surface and (ii) rigid-body reorientation of the entire particle. These two mechanisms act in parallel and either one or the other can dominate, depending on the parameters.

In the case $\left(1 + \frac{2}{\lambda}\right)\frac{D_L}{R^2} \gg D_R$ , the dipolar fluctuations of the enzyme distribution due to surface diffusion are faster than the rigid-body rotational diffusion and they determine the timescale over which the particle transitions from ballistic motion to diffusive motion $\tau^* \approx \frac{1}{\left(1+\frac{2}{\lambda}\right)\frac{D_L}{R^2}}$. Conversely, in the case $D_R \gg \left(1 + \frac{2}{\lambda}\right)\frac{D_L}{R^2}$, the enzymes' diffusion along the surface is slower than the rigid-body rotational diffusion of the particle. In this case, the reorientation of the dipole is mainly due to rigid-body rotations and the characteristic timescale recovers that predicted by the ABP model $\tau^* \approx \frac{1}{D_R}$.

Finally, since the experiments are performed in two dimensions, we restrict the MSD obtained above to the case of motion restricted on a plane. In this case, the MSD obtained above is multiplied by a factor 2/3, see Equation (7) of the next section, which yields:

$$\langle(\mathbf{r}(t) - \mathbf{r}(0))^2\rangle_{2D} = \frac{\lambda\, v_c^2}{3\, D_{eff}^2}\left[e^{-2\,t\,D_{eff}} - 1 + 2\,t\,D_{eff}\right] + 4D_T t. \tag{20}$$

This equation can be readily used to fit the mean square displacement measured in the experiments. It can be further simplified for 2-parameter fitting procedure:

$$\langle(\mathbf{r}(t) - \mathbf{r}(0))^2\rangle_{2D} = A\left[e^{-2\,t\,D_{eff}} - 1 + 2\,t\,D_{eff}\right] + 4D_T t. \tag{21}$$

where $D_T$ can be obtained from the Stokes-Einstein formula:

$$D_T = \frac{k_B T}{6\pi\eta R} \tag{22}$$

We obtained $A = 0.84 \pm 0.14$ and ${D_{eff}}^{-1} = 1.00 \pm 0.12$ for fitting shown in Supplementary Figure 12.

In the next section, numerical simulations of the Brownian motion of each enzyme along the surface of the active particle are presented. The velocity of the particle is calculated from the diffusion of each individual enzyme, which allows to confirm the analytical theory presented here and to explore cases where the number of enzymes is small and fluctuations are large.

# Supplementary Note 2: Stochastic model for motility of a protocell with dynamic membrane

In Appendix S1 we derived an analytical expression for the mean squared displacement (MSD) profile over time. The reader should realize that an individual's particle squared displacement profile will deviate from this mean as a result of stochastic variability. In this Appendix we present a stochastic simulation for the motility of an individual protocell with dynamic membrane. The analytical expression for the MSD can then be verified by comparison to the average of simulated squared-displacement profiles.

## 1 Spherical Brownian motion

Simulation of a spherical Brownian motion (SBM) is key in this work. A SBM starting in $\mathbf{y} \in \mathbb{S}^2(R)$, can be defined by the diffusion equation of the transition density $\mathbf{x} \mapsto \rho_{\mathbf{y}}(\mathbf{x}, t)$ as

$$\frac{\partial \rho_{\mathbf{y}}(\mathbf{x}, t)}{\partial t} = D\nabla^2_{\mathbb{S}^2(R)}\rho_{\mathbf{y}}(\mathbf{x}, t), \rho_{\mathbf{y}}(\mathbf{x}, 0) = \delta(\mathbf{x}, \mathbf{y}), \tag{23}$$

where $\nabla^2_{\mathbb{S}^2(R)}$ is the Laplace-Beltrami operator on the sphere $\mathbb{S}^2(R)$ and $D > 0$ the diffusion coefficient.

We have adopted the simulation strategy presented by Mijatović et al. (2020),[13] but instead we used a normal approximation to simulate from the Wright-Fisher distribution as discussed by Jenkins and Spano (2017).[14] The latter approximation is appropriate since we will simulate for small time steps. Simulation of the SBM for a time step $\Delta t$ is performed by Algorithm 1.

**Algorithm 1.** Simulating increment of a SBM with diffusion coefficient $D$

1: Set time step $\Delta t$, sphere radius $R$ and starting point $\mathbf{y}$
2: Let $\tau = \frac{2D\Delta t}{R^2}$
3: Simulate $M \sim \mathcal{N}(\frac{2\zeta}{\tau}, \frac{2\zeta}{\tau}(\zeta + \beta)^2(1 + \frac{\zeta}{\zeta+\beta} - 2\zeta)\beta^2)$, where $\beta = \frac{\tau}{2}$ and $\zeta = \beta/(\mathrm{e}^{\beta} - 1)$.
4: Simulate $X \sim \mathrm{Beta}\,(1, 1 + M)$
5: Set $O(\mathbf{y}) = I - 2uu^{\top}$, where $u = \frac{((0,0,1)^{\top} - \frac{\mathbf{y}}{R})}{|((0,0,1)^{\top} - \frac{\mathbf{y}}{R})|}$
6: **Return** $R \cdot O(\mathbf{y})(2\sqrt{X(1-X)}Y^{\top}, 1 - 2X)^{\top}$

Examples of three simulated SBMs over the sphere with radius $R = 5$, for 10 seconds, with $D = 0.03, D = 0.1$ and $D = 0.5$ respectively, are presented in Supplementary Fig. 17.

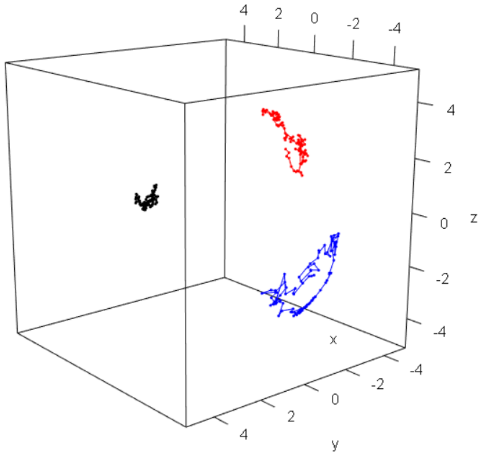


Figure 17: Examples of simulated SBMs on a sphere with radius $R = 5$ and $D = 0.03$ (black), $D = 0.1$ (red) and $D = 0.5$ (blue).

The mean distance traveled from the starting points is 0 as illustrated in Supplementary Fig. 18. The mean squared distance from the starting point does depend on the diffusion coefficient and is illustrated in Supplementary Fig. 18 for $D \in \{0.03, 0.1, 0.5\}$. The signed distance from the starting point is computed as $R \cdot \Delta\sigma \cdot \text{sign}(\phi_2 - \phi_1)$, where $\Delta\sigma$ is the angle between the vector to the starting point and the vector to the new point, $\phi_1$ and $\phi_2$ equal the azimuthal angle of the spherical coordinates of the starting point and the new point respectively. The $\Delta\sigma$ is known as the central angle and can be computed as

$$\Delta\sigma = \arccos(\sin(\phi_1)\sin(\phi_2) + \cos(\phi_1)\cos(\phi_2)\cos(\lambda_2 - \lambda_1)), \tag{24}$$

where $\lambda_1$ and $\lambda_2$ equal the polar angle of the spherical coordinates of the starting point and the new point respectively.

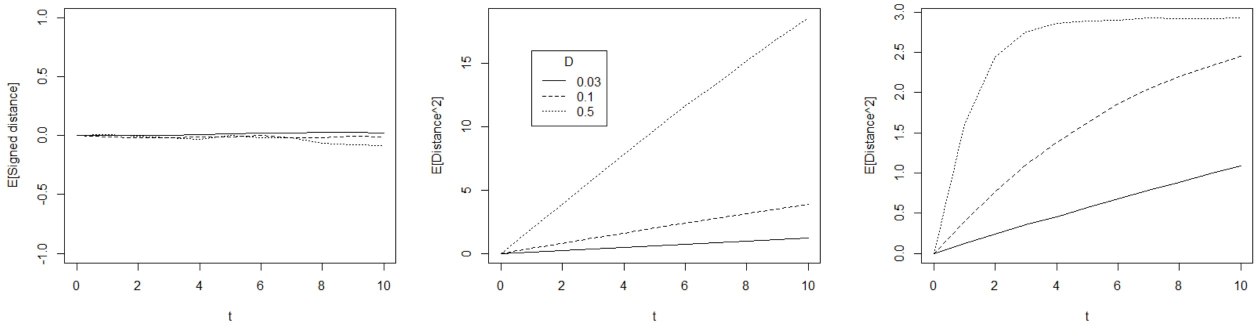


Figure 18: The expected (signed) distance from the starting point (left) and the expected squared distance from the starting point, for diffusion on a sphere with $R = 5$ (middle) or $R = 1$ (right) and $D \in \{0.03, 0.1, 0.5\}$, based on 10000 simulations.

# 2 Motile particle ignoring rotational and lateral diffusion

Let us consider the two-dimensional stochastic process $\mathbf{X}_t$ that represents the position of a motile particle at time $t$ in the $(x, y)$-plane, assuming that there is no rotational diffusion of the particle, nor lateral diffusion of the enzymes attached, i.e.,

$$\mathbf{X}_t = \sqrt{2D_T}\begin{pmatrix} B_x(t) \\ B_y(t) \end{pmatrix} + tv_c\frac{\mathbf{v}_0}{R}, \tag{25}$$

where $B_x(t)$ and $B_y(t)$ independent one-dimensional Brownian motions and $D_T$ represent the translational diffusion coefficient.[12] These Brownian motions model the translational diffusion of the particle. Furthermore,

$$\mathbf{v}_0 = \sum_{i=1}^{N} \mathbf{x}_i, \tag{26}$$

where $N$ equals the random number of enzymes attached to the particle, $\mathbf{x}_i$ equals the position vector of enzyme $i$ projected on the $z = 0$ plane and $v_c$ is a normalization constant that could change with the presence of substrate (fuel). Note that $\frac{\mathbf{v}_0}{R} = \sum_{i=1}^{N} \frac{\mathbf{x}_i}{R}$ then represents the effective-propulsion-direction vector. It is important to realize that it is unknown whether $v_c$ should be negative (motor pushes the particle ) or positive (motor pulls the particle). However, both scenarios will result in the same mean squared displacement (MSD) curve.

The MSD, in the $(x, y)$-plane, over time of such particles equals

$$\mathbb{E}\left[|\mathbf{X}_t|^2\right] = 4D_T t + v_c^2 t^2 \frac{\mathbb{E}\left[\mathbf{v}_0^2\right]}{R^2}. \tag{27}$$

Since $\mathbf{x}_i$ and $\mathbf{x}_j$, for $i \neq j$, are independent and $\mathbb{E}[\mathbf{x}_i] = 0$ due to the symmetry of the particle. By Wald's identity, the expectation of $\mathbf{v}_0^2$ can be written as

$$\mathbb{E}\left[\mathbf{v}_0^2\right] = \lambda\kappa, \tag{28}$$

where $\kappa = \mathbb{E}\left[|\mathbf{x}_i|^2\right]$ and $\lambda = \mathbb{E}[N]$. The projection of velocity vector of the enzyme attached in coordinate $(x, y, z)$ on the sphere to the $z = 0$ plane equals $(x, y)$. Under the assumption that the particle is functionalized randomly, we know that

$$\kappa = \int_{-R}^{R} \left(R^2 - z^2\right) \frac{2\pi(R - z)}{4\pi R^2} \mathrm{d}z = \frac{2}{3} R^2. \tag{29}$$

Thus,

$$\mathbb{E}\left[|\mathbf{X}_t|^2\right] = 4D_T t + v_c^2 t^2 \frac{2}{3}\lambda. \tag{30}$$

We have simulated 1000 times $\mathbf{X}_t$ for 10 seconds, where we have assumed $R = 1$, $v_c = 1$, $\sqrt{2D_T} = 1$, $N \sim Poi(\lambda)$ for $\lambda \in \{20, 100, 1000\}$. The MSD in the $(x, y)$-plane of these simulations, as well as the theoretical curve in (30), are presented in Supplementary Fig. 19 and do match. It is important to remark that the MSD curve is not unique if $D_T$ is given and depends on the product of $v_c^2$ and $[N]$, i.e. lowering the expected enzyme density with a factor $c$ results in the same MSD curve if $v_c$ is increased by a factor $\sqrt{c}$.

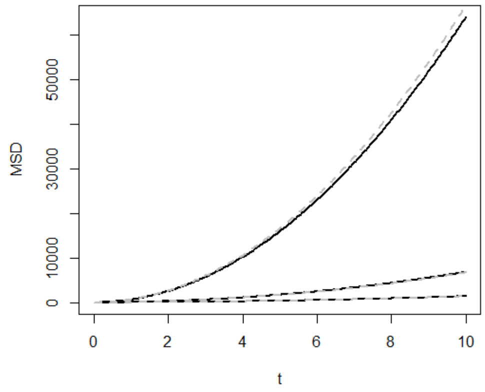


Figure 19: MSD for a particle ignoring lateral diffusion of the enzymes and rotational diffusion of the particle. Curves are presented for $\lambda \in \{20, 100, 1000\}$ (black), where the MSD increases with the expected enzyme density. Theoretical curves are shown as dotted gray lines and nicely overlay with the means derived from the simulation ($n = 1000$).

## 2.1 $D_T$ estimates

The diffusion of small ($R = 0.6$um) and large ($R = 1.8$um) particles has been measured experimentally in the absence of fuel (see Materials). For those particles the MSD equals $4D_T t$, which allows us to estimate $D_{T,0.6}$ and $D_{T,1.8}$. By fitting a simple (no intercept) linear regression we estimated translational diffusion coefficients equal $D_{T,0.6} = 0.236$ (0.0015) and $D_{T,1.8} = 0.079$ (0.0018) respectively. These diffusion coefficients are known to equal

$$\frac{k_B T}{6\pi\eta}\frac{1}{R}, \tag{31}$$

where $k_B T$ is the thermal energy and $\eta$ is the viscosity of the medium in which the particle diffuses.[12] All experiments have been performed in similar conditions such that we can assume $\frac{k_B T}{6\pi\eta}$ to be constant. Based on the $D_{T,0.6}$ and $D_{T,1.8}$ values we estimate this constant as $0.236 \cdot 0.6 = 0.141$ and $0.079 \cdot 1.8 = 0.142$ respectively. Therefore we will assume that $\frac{k_B T}{6\pi\eta} = 0.14$ in the rest of this study.

# 3 Motile particle with lateral diffusive enzymes

We further extend the model to describe the stochastic process $\mathbf{Y}_t$ that represents the position of a motile particle at time $t$ in the $(x, y)$-plane, where enzymes (motors) additionally undergo lateral diffusion, and is modeled as

$$Y_t = \sqrt{2D_T}\begin{pmatrix} B_x(t) \\ B_y(t) \end{pmatrix} + v_c \sum_{j=0}^{n_t} \frac{1}{R}\mathbf{v}_{j \cdot dt}, \tag{32}$$

where $dt$ equals the size of the time steps of the random walks and $\mathbf{v}_{j \cdot dt}$ is the net-propulsion vector, in the $(x, y)$-plane, as a result of the enzymes at time $j \cdot dt$ such that $\mathbf{v}_{x,j \cdot dt} = \sum_{i=1}^{N} \mathbf{x}_{i,j \cdot dt}$. This net propulsion is not constant as a result of the lateral movement of the enzymes. We assume each enzyme to undergo an independent SBM with diffusion coefficient $D_L$ over the surface of the sphere (see Section 1 for a description). Notice that the speed of the diffusion is independent of the size of the particle; two examples of the movement of an enzyme on a particle with $R = 0.6$ and $R = 1.8$ respectively, for $D_L = 0.03$ are illustrated in Supplementary Fig. 20.

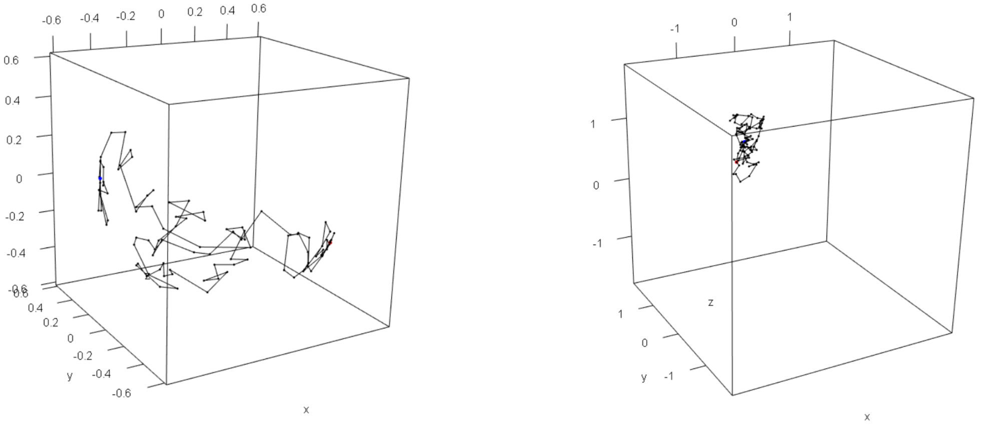


Figure 20: Diffusion of an enzyme during 10 seconds and $D_L = 0.03$ on a spherical particle with $R = 0.6$ (left) and on a particle with $R = 1.8$ (right).

## 3.1 FRAP experiment

To validate how accurate this SBM describes the lateral diffusion of the enzymes we have mimicked the fluorescence recovery after photobleaching (FRAP) experiment that was performed in the lab. Firstly, we have randomly 'functionalized' a spherical particle with $R = 5.0$ using a Poisson-distributed ($\lambda = 10000$) number of enzymes $N$. Subsequently, part of the enzymes are hit with a 'laser' with radius 1.25nm centered around $(\sqrt{5^2 - 1.25^2}\sin(\alpha), \sqrt{5^2 - 1.25^2}\cos(\alpha))$, where

$$\alpha = \text{acos}\left(\frac{\sqrt{(5^2 - 1.25^2)}}{5}\right). \tag{33}$$

From the FRAP experiment performed in the lab it was observed that roughly a fraction of 35% of the enzymes within the $x - y$ area that the laser hit were not photo-bleached. Therefore, we have independently removed each enzyme within the $(x - y)$ laser area with a probability 0.65, see Supplementary Fig. 21. Then we count the variable number (due to the lateral diffusion) of enzymes within the bleached region, and within 1 micron of the focal plane $z \in (-0.5, 0.5)$, over time, and compared this number to a reference region of the same size at the other side of the particle. In Supplementary Fig. 21 we also show the situation after 5 seconds.

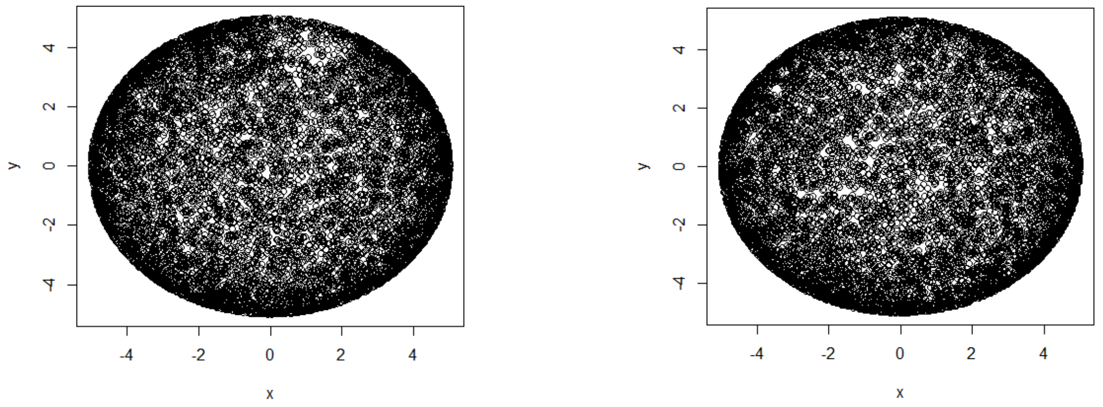


Figure 21: Particle direct after photo-bleaching ($t = 0$, left, where we observe a bright 'hole' in the top left side) and particle after five seconds of enzyme diffusion ($t = 5$, right, where the 'hole' has diffused away)

From the experiment $D_L$ was estimated as 0.035 for the catalase (mCAT) enzymes. The intensity curve obtained from simulation with this particular value of $D_L$ was compared to the observed intensity curves from the real FRAP experiments as shown in Supplementary Fig. 22. We conclude that the SBM model can be used accurately to describe the lateral diffusion of enzymes. The mean squared displacement from the starting point of an enzyme for $t \in [0, 10]$ with $D_L = 0.035$ was presented in Supplementary Fig. 18.

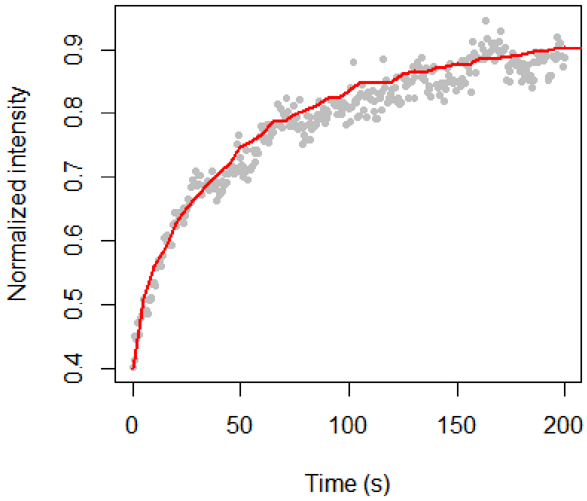


Figure 22: Intensity measurements from the (real) FRAP experiment (grey dots), and the average of 1000 simulated intensity measurements over time for $D_L = 0.035$ (red).

### 3.2 MSD profile

Although the position vectors of one specific enzyme are strongly correlated over time, the position vectors of different enzymes are still independent over time. As a result the MSD can be written as

$$\mathbb{E}\left[|\mathbf{Y}_t|^2\right] = 4D_T t + \frac{v_c^2}{R^2}\mathbb{E}\left(\Big|\sum_{j=0}^{n_t}\sum_{i=1}^{N}\mathbf{x}_{i,j\cdot dt}\Big|^2\right) \tag{34}$$

$$= 4D_T t + \frac{v_c^2}{R^2}\lambda\mathbb{E}\left(\Big|\sum_{j=0}^{n_t}\mathbf{x}_{i,j\cdot dt}\Big|^2\right).$$

In Supplementary Fig. 23 we present the MSD profiles from simulations of particles with $R \in \{0.6, 1, 1.8\}$, $\lambda = 100$ and $D_L \in \{0, 0.03, 0.1, 0.25, 0.9, 3\}$.

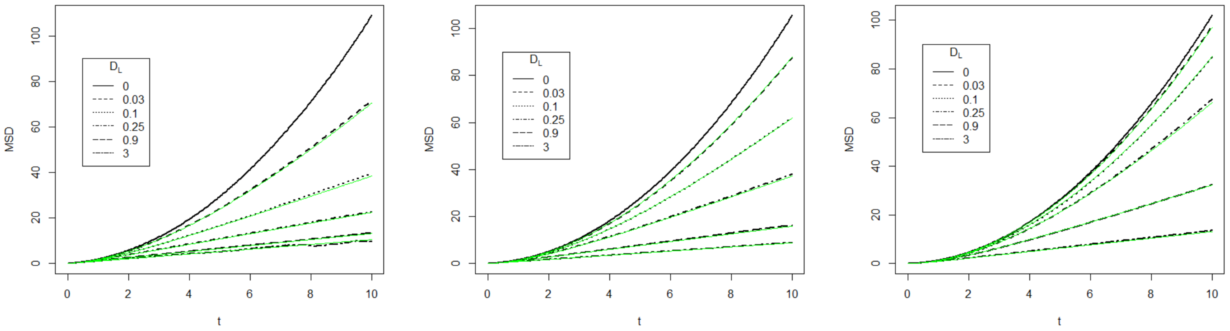


Figure 23: MSD curve for a particle with radius $R = 0.6$ (left), $R = 1$ (middle) or $R = 1.8$ (right), $\lambda = 100$, $v_c = 1/\sqrt{\frac{2}{3}\lambda} = 0.122$, $D_T = \frac{0.14}{R}$ and different $D_L$ (see legend), while ignoring rotational diffusion, based on $n = 10000$ simulations. The green lines represent the analytical expression (35) derived in this study for $V = 1$, $D_R = 0$ and varying $D_L$.

In this work (see Appendix S1) we derived an analytical form for (34),

$$\frac{1}{2}\left(\frac{v_c\sqrt{\frac{2}{3}\lambda}}{D_{\text{eff}}}\right)^2\left(e^{-2D_{\text{eff}}t} + 2tD_{\text{eff}} - 1\right) + 4D_T t, \tag{35}$$

where $D_{\text{eff}} = D_R + (1 + \frac{2}{\lambda})\frac{D_L}{R^2}$ and $D_R$ equals the rotational diffusion coefficient which will be discussed in Section 4. For $D_R = 0$, Supplementary Fig. 23 confirms the validity of this expression in our simulation study. For the experimental particles studied $\lambda > 5000$ as a result of which $D_{\text{eff}}$ can be approximated by $D_R + \frac{D_L}{R^2}$. Then, the MSD profile does only depend on $\lambda$ via $v_c\sqrt{\lambda}$, for which reason we can simulate with a lower number of $\lambda$ and a higher value of $v_c$ to seriously reduce the computation time.

## 4 Motile particles with lateral diffusive enzymes and rotational diffusion

Finally, we extend our model for the motile particle by accounting for the rotational diffusion. The rotational diffusion is modeled by rotation of the coordinate system. The rotation of the coordinate system follows the rotation of the vector $(0, 0, 1)^\top$ that undergoes a SBM on the unit sphere characterized with $D = D_R$, where $D_R = \frac{k_B T}{8\pi\eta}\frac{1}{R^3}$, referred to as $\mathbf{b}_t$. Subsequently, the net-velocity vector at time $t$, is rotated following this SBM as described by Algorithm 2.

**Algorithm 2** Simulating rotational diffusion with diffusion coefficient $D$.

1: Simulate $\mathbf{b}_t$ according to Algorithm 1 using $D = D_R$.
2: Let $\mathbf{v}_t$ be equal to the net-velocity vector of the particle without rotational diffusion and let $|\mathbf{v}_t| = n_t$.
3: Let $\mathbf{z}_t$ be equal to the unit directional vector such that $n_t\mathbf{z}_t = \mathbf{v}_t$.
4: Set $O(\mathbf{z}_t) = I - 2uu^\top$, where $u = \frac{((0,0,1)^\top - \mathbf{z}_t)}{|((0,0,1)^\top - \mathbf{z})|}$.
5: **Return** $\mathbf{v}_t^{'} = n_t \cdot O(\mathbf{z}_t)\mathbf{b}_t$.

As $D_R = \frac{k_{\rm B}T}{8\pi\eta}\frac{1}{R^3}$, large particles rotate slower than small particles.[12] Due to the rotations the positions of the different individual enzymes over time become correlated. The MSD can be written as

$$\mathbb{E}\left[|\mathbf{Y}_t|^2\right] = 4D_T t + \left(\frac{v_c}{R}\right)^2 \mathbb{E}\left[\left|\sum_{j=0}^{n_t}\sum_{i=1}^{N}\mathbf{x}_{i,j\cdot dt}\right|^2\right], \tag{36}$$

and the analytical form has been presented as Equations(35). In Supplementary Fig. 24 we present the influence of $D_R$ on the the MSD presented in (36), for particle with $R \in \{0.6, 1, 1.8\}$, where $\lambda = 100$ (and $\sqrt{2D_T} = 1$), both for $D_L = 0.03$ and $D_L = 0$. Again we have validated the accuracy of the analytical MSD expression (35), presented as the green lines in Supplementary Fig. 24.

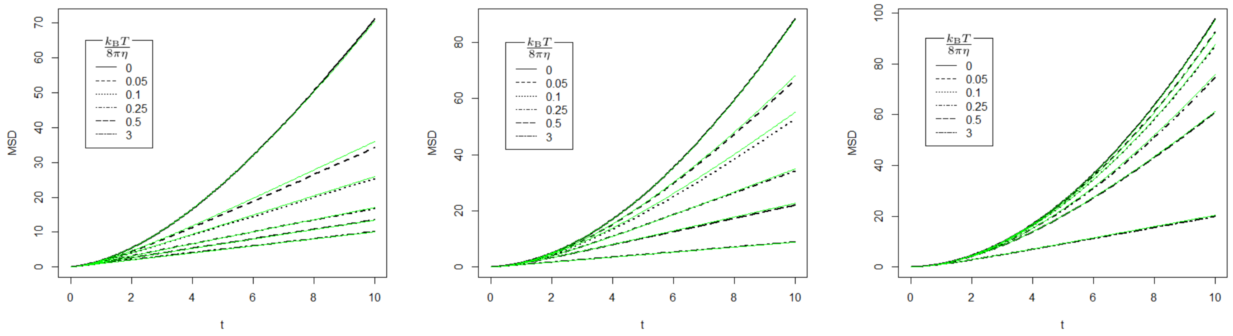


Figure 24: MSD curve for a particle with radius $R = 0.6$ (left), $R = 1$ (middle) or $R = 1.8$ (right), $\lambda = 100$, $v_c = 0.122$, $D_L = 0.03$, $D_T = \frac{0.14}{R}$ and different $\frac{k_{\rm B}T}{8\pi\eta}$ (see legend), based on $n = 10000$ simulations. The green lines represent the analytical expression (35) derived in this study for the MSD.

Furthermore, in Supplementary Fig. 25, where $D_L = 0$, we have also plotted a re-parameterized form of the expression as derived by Howse et al. (2007),[12] by substitution of $D_L = 0$ in (35):

$$\frac{1}{2}\left(\frac{v_c\sqrt{\frac{2}{3}\lambda}}{D_R}\right)^2 \left(e^{-2D_R t} + 2tD_R - 1\right) + 4D_T t. \tag{37}$$

The theoretical expression (37) does indeed fit the MSD curves derived with our SBM model for the rotation diffusion, from which we conclude that the latter was indeed an appropriate approach.

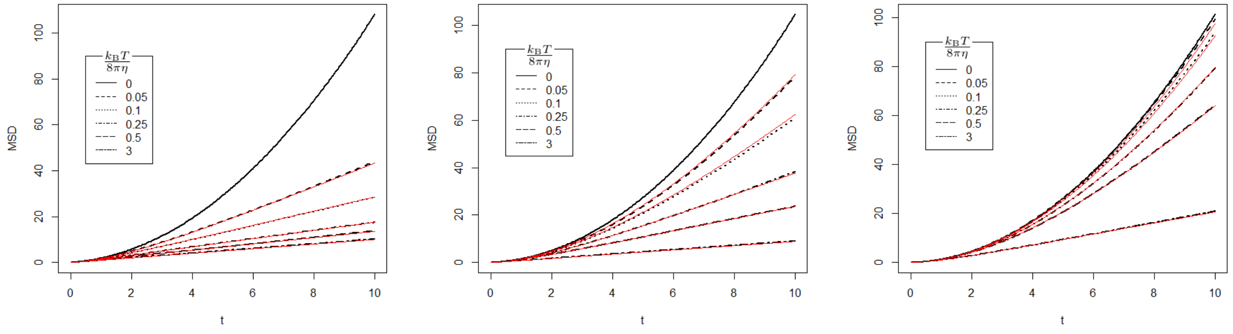


Figure 25: MSD curve for a particle with radius $R = 0.6$ (left), $R = 1$ (middle) or $R = 1.8$ (right), $\lambda = 100$, $v_c = 0.122$, $D_L = 0$, $D_T = \frac{0.14}{R}$ and different $\frac{k_BT}{8\pi\eta}$ (see legend), based on $n = 10000$ simulations. The red lines represent the analytical expression in absence of lateral diffusion as presented in (37).

# 5 Experimental data fitting and simulation

Based on the no-fuel data we did derive $\frac{k_BT}{6\pi\eta}$ as 0.14 as a result of which $D_{T,0.6} = 0.233$ and $D_{T,1.8} = 0.078$. The rotational diffusion coefficient is known to equal $\frac{k_BT}{6\pi\eta}\frac{3}{4}\frac{1}{R^3}$ such that $D_{R,1.8} = 0.018$ and $D_{R,0.6} = 0.486$. Setting-specific estimates of $V = v_c\sqrt{\frac{2}{3}\lambda}$ and $D_L$ can be derived by fitting Equation (35) to each setting-specific dataset. However, several settings have shared parameters, e.g. $V$ in cross-linked and non-cross-linked particles. Therefore we fit the joint datasets of the large particles (both cross-linked and non-cross-linked) with

$$4D_{T,1.8}t + 0.5\left(\frac{V_{1.8}}{D_{R,1.8} + \mathbb{1}_{\text{ncs}}\frac{D_{L,1.8}}{1.8^2}}\right)^2\left(e^{-2(D_{R,1.8}+\mathbb{1}_{\text{ncs}}\frac{D_{L,1.8}}{1.8^2})t} + 2t(D_{R,1.8} + \mathbb{1}_{\text{ncs}}\frac{D_{L,1.8}}{1.8^2}) - 1\right), \quad (38)$$

where $\mathbb{1}_{\text{ncs}}$ presents an indicator function that equals one for non-crosslinked-particles. Similarly, we fit the joint datasets of the small particles (different enzymes, different densities, cross-linked and non-crosslinked) with

$$4D_{T,0.6}t+$$
$$0.5\left(\frac{\sum_{x\in\mathcal{X}}\mathbb{1}_x V_{0.6,x}}{D_{R,0.6} + \mathbb{1}_{\text{ncs}}(\sum_{y\in\mathcal{Y}}\mathbb{1}_y\frac{D_{L,0.6,y}}{0.6^2})}\right)^2\left(e^{-2t(D_{R,0.6}+\mathbb{1}_{\text{ncs}}(\sum_{y\in\mathcal{Y}}\mathbb{1}_y\frac{D_{L,0.6,y}}{0.6^2}))} + 2t(D_{R,0.6} + \mathbb{1}_{\text{ncs}}(\sum_{y\in\mathcal{Y}}\mathbb{1}_y\frac{D_{L,0.6,y}}{0.6^2})) - 1\right), \quad (39)$$

where $\mathcal{X} = \{$low-mCAT, medium-mCAT, high-mCAT, low-mUR, medium-mUR, high-mUR$\}$ and $\mathcal{Y} = \{$mCAT, mUR$\}$. The fitting is performed based on non-linear least-squares estimation using the `nlsLM()` function, from the package `minpack.lm` in [R]. Based on the FRAP experiments we use the restrictions $D_{\text{L},0.6,\text{mCAT}} \geq 0.036$ and $D_{\text{L},0.6,\text{mUR}} \geq 0.03$. The parameter estimates, standard errors and predicted MSD curves can be found in Supplementary Fig. 26.

| Parameter | Estimate | SE |
|---|---|---|
| $V_{1.8}$ | 0.576 | 0.002 |
| $D_{\mathrm{L},1.8}$ | 0.917 | 0.030 |
| $V_{0.6,\text{low-mCAT}}$ | 0.587 | 0.025 |
| $V_{0.6,\text{med-mCAT}}$ | 0.964 | 0.009 |
| $V_{0.6,\text{high-mCAT}}$ | 0.754 | 0.020 |
| $D_{\mathrm{L},0.6,\text{mCAT}}$ | 0.036 | 0.009 |
| $V_{0.6,\text{low-mUR}}$ | 0.470 | 0.052 |
| $V_{0.6,\text{med-mUR}}$ | 0.985 | 0.098 |
| $V_{0.6,\text{high-mUR}}$ | 0.295 | 0.051 |
| $D_{\mathrm{L},0.6,\text{mUR}}$ | 0.030 | 0.062 |

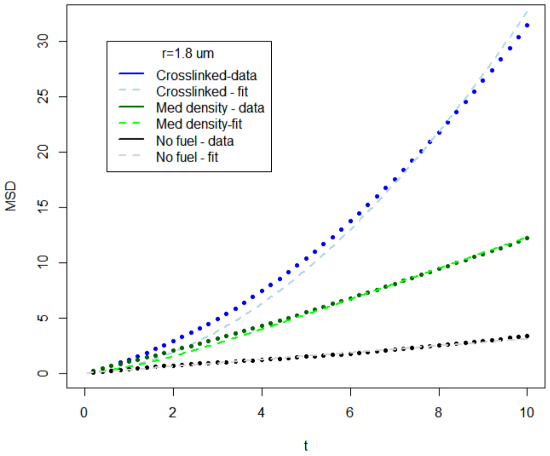


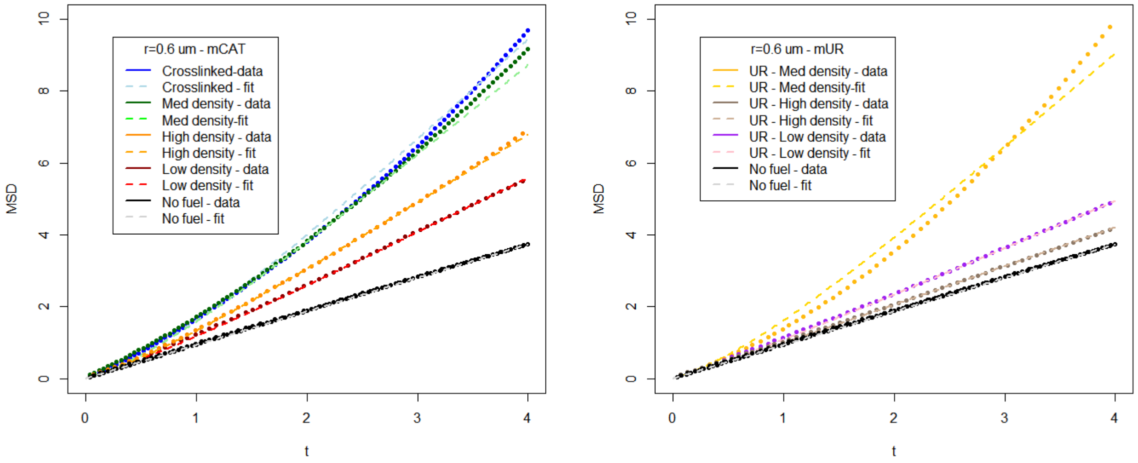


Figure 26: Parameter estimates, experimentally observed MSD and predicted MSD for the different settings studied.

## 5.1 Simulation

Using the parameters obtained from the experimental data we can now simulate all types of particles that have been studied. The MSD curves of the simulations for the different settings are presented together with the experimentally observed MSD curves in Figures 27, 28 and 29. It is important to remark that with 10000 simulations the mean of the simulated particles is equal to the analytical fit presented in Supplementary Fig. 26.

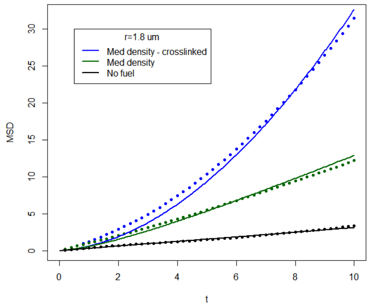

Figure 27: MSD obtained from experiments for large particles functionalized with catalase enzymes (dots) and the MSD based on 10000 simulated equivalents.

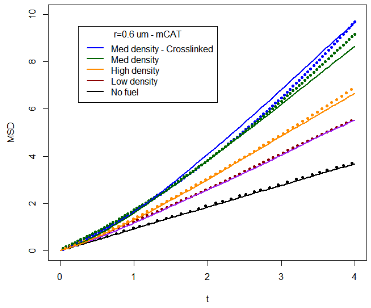

Figure 28: MSD obtained from experiments for small particles functionalized with catalase enzymes (dots) and the MSD based on 10000 simulated equivalents.

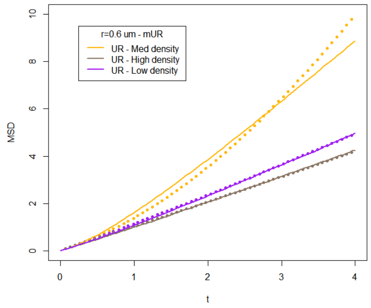

Figure 29: MSD obtained from experiments for small particles functionalized with urease enzymes (dots) and the MSD based on 10000 simulated equivalents.

Thus, it becomes clear that we can appropriately describe the experimental observations with the model proposed. To do so, the lateral diffusion coefficient in the case of the large mCAT particles should substantially differ from the lateral diffusion coefficient in the case of the small particles.

# 6 Instantaneous velocity

For the simulated particles from Section 5.1 the 'instantaneous' velocity was derived over time as

$$\frac{\sqrt{|\mathbf{Y}_{t+1} - \mathbf{Y}_{t-1}|}}{2dt}. \tag{40}$$

The distribution of these instantaneous velocities are presented as Supplementary Fig. 30.

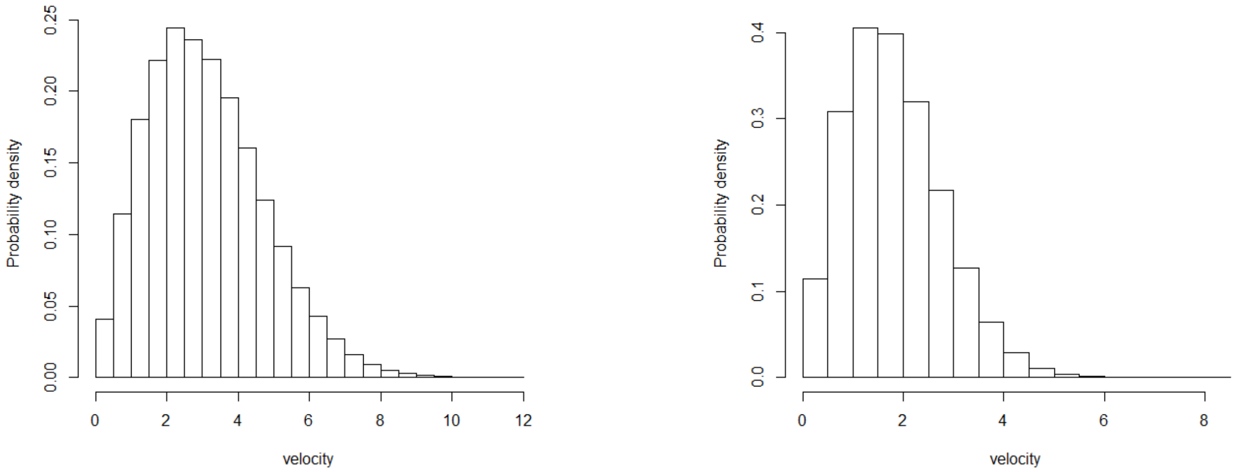

Figure 30: Distribution of instantaneous velocity derived from 10000 simulated small (left) and large (right) cross-linked particles

The mean velocity of the simulated large particles (1.8 um/s) deviates from the mean velocity of the large experimental (non-crosslinked) particles (3.24 um/s). The velocity highly depends on the size of the translational diffusion coefficient $D_T$. On the contrary, the influence of $D_T$ on the MSD was only minor for the large particles, such that the fits to the data (with fuel, Supplementary Fig. 27) were insensitive to the value of $D_T$. In Supplementary Fig. 31 the cumulative probability distribution of the experimentally observed velocities

is presented together with the distribution of simulated large particles using $\frac{k_B T}{6\pi\eta} = 0.45$, $V_{1.8} = 0.576$ and $D_{L,1.8} = 0.917$. For this simulation setting the mean velocity (3.18 um/s) corresponds to the experimentally observed average. It is important to note that the experimentally observed velocities are discretized as a result of the finite pixel size used during the imaging. We conclude that the variability in the velocities is well explained by the stochastic model. However, it becomes clear that a fraction of approximately 1% of the observed velocities is too extreme ($> 12.5$ um/s) to be explained by our stochastic model, as these velocities are never observed in the simulations.

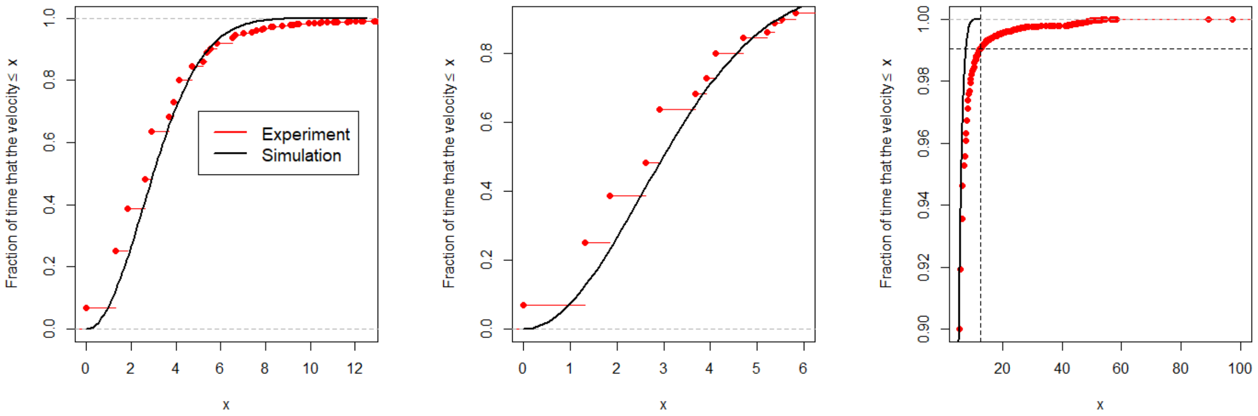


Figure 31: Cumulative distribution function, i.e. fraction of the time that the velocity is less than or equal to $x$, of the instantaneous velocity (left) observed experimentally (red) and derived from 10000 simulated (10s) large particles (black). Adjusted $x$- and $y$- axis are presented to emphasize the agreement (middle) and difference (right) of the two distributions.

The 15 large coacervates from the experiment were moving at such an extreme velocity ($> 12.5$ um/s) 0% (9 particles), 1% (5 particles), 3% (1 particle) and 6% (1 particle) of the time respectively. For this reason we state that the latter two particles showed 'run-and-tumble behavior' (see main text).

In summary, we can conclude that we can explain the motile behavior of the vast majority of the particles. In small-sized particles the velocities are well described by the stochastic model. For large particles, 10% of the large coacervates modified with catalase show a run and tumble behaviour (Movie S6) that far exceeds the velocity distribution and cannot be explained by this model. We assume that a more complex combination of stochastic processes is at the core of this yet unexplained variability; for example due to interactions between the enzymes.

# Supplementary References:


1 Mason, A. F., Buddingh', B. C., Williams, D. S. & van Hest, J. C. Hierarchical self-assembly of a copolymer-stabilized coacervate protocell. *J. Am. Chem. Soc.* **139**, 17309-17312,(2017).

2 Couffin, A., Delcroix, D., Martín-Vaca, B., Bourissou, D. & Navarro, C. Mild and efficient preparation of block and gradient copolymers by methanesulfonic acid catalyzed ring-opening polymerization of caprolactone and trimethylene carbonate. *Macromolecules* **46**, 4354-4360,(2013).

3 Zou, J. *et al.* A facile glovebox-free strategy to significantly accelerate the syntheses of well-defined polypeptides by N-carboxyanhydride (NCA) ring opening polymerizations. *Macromolecules* **46**, 4223-4226,(2013).

4 Axelrod, D., Koppel, D., Schlessinger, J., Elson, E. & Webb, W. W. Mobility measurement by analysis of fluorescence photobleaching recovery kinetics. *Biophys. J.* **16**, 1055-1069,(1976).

5 Phair, R. D. & Misteli, T. Kinetic modelling approaches to in vivo imaging. *Nat. Rev. Mol. Cell Biol.* **2**, 898,(2001).

6 Gosting, L. J. & Akeley, D. F. A Study of the Diffusion of Urea in Water at 25° with the Gouy Interference Method. *J. Am. Chem. Soc.* **74**, 2058-2060,(1952).

7 Axelsson, I. Characterization of proteins and other macromolecules by agarose gel chromatography. *J. Chromatogr. A* **152**, 21-32,(1978).

8 Follmer, C., Pereira, F. V., Da Silveira, N. P. & Carlini, C. R. Jack bean urease (EC 3.5.1.5) aggregation monitored by dynamic and static light scattering. *Biophys. Chem.* **111**, 79-87,(2004).

9 Ortiz de Zárate, J. M. & Sengers, J. V. *Hydrodynamic fluctuations in fluids and fluid mixtures*. (Elsevier, Amsterdam, 2006).

10 Doi, M. & Edwards, S. F. *The Theory of Polymer Dynamics*. (Clarendon Press, New York, 1988).

11 Dhont, J. K. *An introduction to dynamics of colloids*. (Elsevier, Amsterdam, 1996).

12 Howse, J. R. *et al.* Self-motile colloidal particles: from directed propulsion to random walk. *Phys. Rev. Lett.* **99**, 048102,(2007).

13 Mijatović, A., Mramor, V. & Bravo, G. U. A note on the exact simulation of spherical Brownian motion. *Stat. Probab. Lett.* **165**, 108836,(2020).

14 Jenkins, P. A. & Spano, D. Exact simulation of the Wright–Fisher diffusion. *Ann. Appl.Probab*. **27**, 1478-1509,(2017).
